# Preservation of exopolymeric substances in estuarine sediments

**DOI:** 10.3389/fmicb.2022.921154

**Published:** 2022-08-18

**Authors:** Thibault Duteil, Raphaël Bourillot, Olivier Braissant, Brian Grégoire, Maud Leloup, Eric Portier, Benjamin Brigaud, Hugues Féniès, Isabelle Svahn, Adrien Henry, Yusuke Yokoyama, Pieter T. Visscher

**Affiliations:** ^1^Univ. Bordeaux, CNRS, Bordeaux INP, EPOC, UMR 5805, Pessac, France; ^2^Department Biomedical Engineering (DBE), Center for Biomechanics and Biocalorimetry, University of Basel, Allschwil, Switzerland; ^3^Institut de Chimie des Milieux et Matériaux de Poitiers (IC2MP), Centre National de la Recherche Scientifique (CNRS), Université de Poitiers, Poitiers, France; ^4^45-8 Energy, Metz, France; ^5^CNRS, GEOPS, Université Paris-Saclay, Orsay, France; ^6^Bordeaux Imaging Center (BIC), CNRS, Université de Bordeaux, Bordeaux, France; ^7^Department of Earth and Planetary Sciences, Atmosphere and Ocean Research Institute, University of Tokyo, Kashiwanoha, Chiba, Japan; ^8^Department of Marine Sciences and Geosciences, University of Connecticut, Groton, CT, United States; ^9^CNRS, Biogéosciences, Université de Bourgogne Franche-Comté, Dijon, France

**Keywords:** estuarine sediments, diatom biofilms, exopolymeric substances, FTIR – spectroscopy, cryo-SEM, sedimentary core, EPS-sediment aggregates, preservation

## Abstract

The surface of intertidal estuarine sediments is covered with diatom biofilms excreting exopolymeric substances (EPSs) through photosynthesis. These EPSs are highly reactive and increase sediment cohesiveness notably through organo-mineral interactions. In most sedimentary environments, EPSs are partly to fully degraded by heterotrophic bacteria in the uppermost millimeters of the sediment and so they are thought to be virtually absent deeper in the sedimentary column. Here, we present the first evidence of the preservation of EPSs and EPS-mineral aggregates in a 6-m-long sedimentary core obtained from an estuarine point bar in the Gironde Estuary. EPSs were extracted from 18 depth intervals along the core, and their physicochemical properties were characterized by (i) wet chemical assays to measure the concentrations of polysaccharides and proteins, and EPS deprotonation of functional groups, (ii) acid–base titrations, and (iii) Fourier transform infrared spectroscopy. EPS-sediment complexes were also imaged using cryo-scanning electron microscopy. EPS results were analyzed in the context of sediment properties including facies, grain size, and total organic carbon, and of metabolic and enzymatic activities. Our results showed a predictable decrease in EPS concentrations (proteins and polysaccharides) and reactivity from the surface biofilm to a depth of 0.5 m, possibly linked to heterotrophic degradation. Concentrations remained relatively low down to *ca.* 4.3 m deep. Surprisingly, at that depth EPSs abundance was comparable to the surface and showed a downward decrease to 6.08 m. cryo-scanning electron microscopy (Cryo-SEM) showed that the EPS complexes with sediment were abundant at all studied depth and potentially protected EPSs from degradation. EPS composition did not change substantially from the surface to the bottom of the core. EPS concentrations and acidity were anti-correlated with metabolic activity, but showed no statistical correlation with grain size, TOC, depth or enzymatic activity. Maximum EPS concentrations were found at the top of tide-dominated sedimentary sequences, and very low concentrations were found in river flood-dominated sedimentary sequences. Based on this observation, we propose a scenario where biofilm development and EPS production are maximal when (i) the point bar and the intertidal areas were the most extensive, i.e., tide-dominated sequences and (ii) the tide-dominated deposit were succeeded by rapid burial beneath sediments, potentially decreasing the probability of encounter between bacterial cells and EPSs.

## Introduction

Diatoms are a major constituent of microphytobenthic biofilms covering intertidal or shallow subtidal coastal and estuarine sediments (Smith and Underwood, 1998). These biofilm communities excrete copious amounts of exopolymeric substances (EPSs), forming a hydrated matrix between the cells and the sediments (De Winder et al., 1999). By secreting EPSs, benthic diatoms establish a microenvironment that protects cells against desiccation, allows motility through the sediment, and supports communication and exchange of metabolites among cells (Underwood and Paterson, 2003; Decho and Gutierrez, 2017). Diatom EPSs are composed of 40 to 90% polysaccharides, but also include proteins, nucleic acids, lipids, and low-molecular weight, non-carbohydrate compounds such as pyruvate and succinate (Underwood and Paterson, 2003). The negatively charged reactive functional groups provide the EPS a cation binding capacity to sequester metal ions or metalloids (Braissant et al., 2007). EPS also possess electrostatic, hydrophilic and hydrophobic properties allowing their sorption to mineral surfaces (Decho, 2000; Duteil et al., 2020). As a consequence, EPSs promote the cohesiveness and stability of the sediment (Tolhurst et al., 2002), impacting bedform morphology (De Winder et al., 1999; Malarkey et al., 2015). In estuaries, EPSs also bind clay and sand particles, resulting in the formation of thin clay envelopes, also referred to as detrital clay coats, that surround sand grains (Wooldridge et al., 2017; Virolle et al., 2019b, 2021; Duteil et al., 2020).

The production and consumption of EPSs in subsurface sediments, as well as their composition and properties (e.g., negatively charged groups), are poorly understood. EPSs are mainly produced by photoautotrophic microorganisms at the surface (e.g., cyanobacteria, purple- and green sulfur bacteria, benthic diatoms; Taylor et al., 2013). Below the sediment photic zone, heterotrophic communities (e.g., sulfate-reducing bacteria) are also able to produce EPSs, albeit at lower production rates than photosynthetic communities (Braissant et al., 2007).

The amount of EPSs typically decreases in the first millimeters to centimeters of estuarine mudflats (Paterson et al., 2000), but also in hypersaline stromatolites and microbial mats (Decho et al., 2005; Braissant et al., 2009; Pace et al., 2018), riverine sediments (Gerbersdorf et al., 2009a), or dryland cyanobacterial crusts (Mager, 2010). This decrease could result from their consumption by heterotrophic microorganisms. While labile forms of EPSs such as low molecular weight components are preferentially consumed by heterotrophs, refractory high molecular weight polymers (e.g., proteins) have a higher preservation potential (Smith and Underwood, 1998; Kleber et al., 2007). The preservation of EPSs in the sediment could be enhanced by various mechanisms, e.g., (i) inherent recalcitrance of specific EPS moieties against enzymatic microbial degradation, (ii) chemical stabilization by interactions between minerals and reactive groups in EPSs, (iii) physical protection through the formation of aggregates with minerals (Kleber et al., 2015) or (iv) reduced microbial enzymatic activity related to the dissolution of organic substrates (Traving et al., 2015). However, it is not well understood to which degree the EPSs persist with depth in sediments (Kleber et al., 2015).

In this study, we analyzed EPSs extracted from a 6 m-long sedimentary core drilled in an estuarine point bar in the Gironde Estuary (southwest France). The composition including the physicochemical properties of EPSs are determined at several depths by a combination of wet chemical assays, acid–base titrations, Fourier Transform-Infrared spectroscopy (FT-IR), and cryo-electron microscopy. EPS characteristics are analyzed in the context of microbial activity (enzymatic and metabolic rates), sediment properties (e.g., grain size, total organic carbon) and sedimentary facies (e.g., tidal and river flood deposits), in order to understand the factors potentially controlling EPS production and preservation in estuaries.

### Sedimentological settings

**Figure 1:**
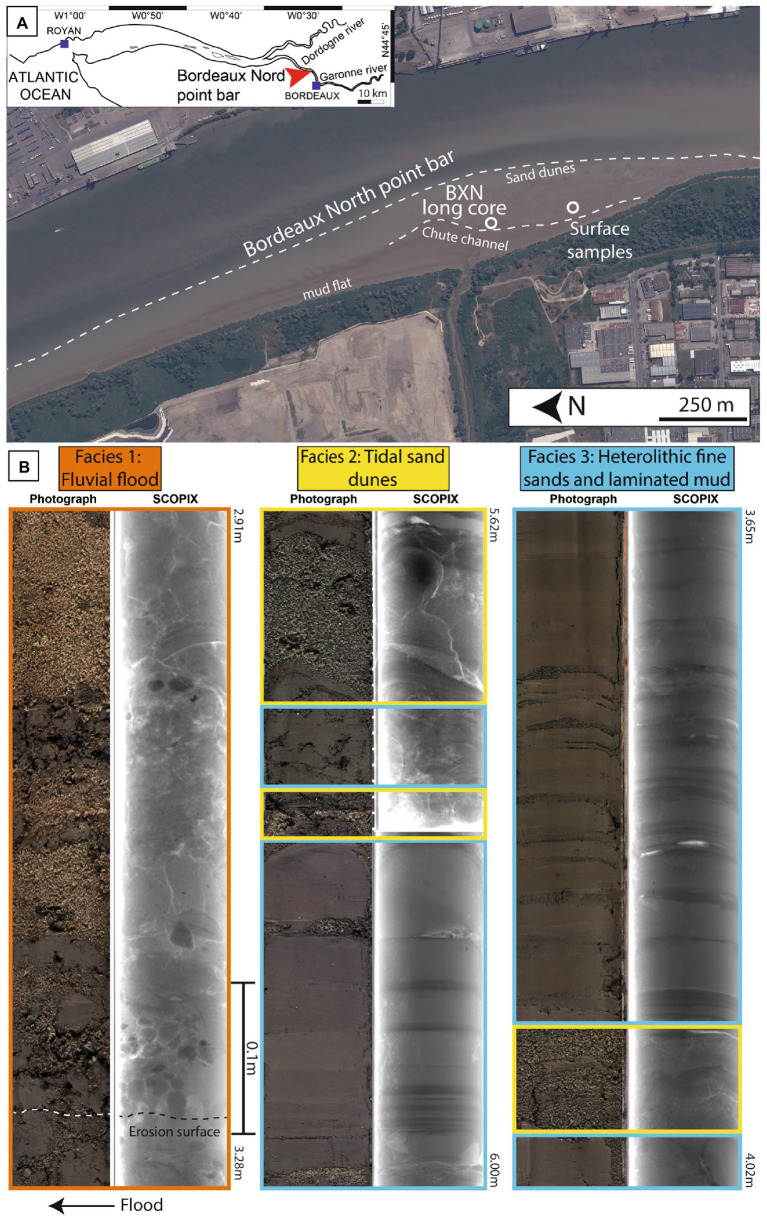
**(A)** Map of the Gironde estuary showing the location of the Bordeaux North point bar, and satellite image of the point bar at low tide (Google Earth Pro) showing the location of the studied core (BXN Long Core) and surface samples. Three sedimentary domains are visible in the intertidal zone of the point bar: the mud flat, the chute channel, and the sand dunes. **(B)** Photographs and *X*-Ray image (SCOPIX) of the principal facies found in the BXN long core.

The Bordeaux North point bar is located in the Garonne estuarine channel, 94 km upstream from the estuary mouth. The accretion of this point bar was probably started less than 300 years ago (Virolle et al., 2021). The bar is 1.1 km long (north–south axis) and 0.2 km wide (east–west axis). The Gironde Estuary has a turbidity maximum zone (TMZ) that forms a dynamic turbid cloud in the water column (Sottolichio et al., 2011). The TMZ has a high concentration of suspended particulate matter (concentration between 1 and 10 g L^−1^), spreading for 70 to 80 km along the estuary (Allen and Castaing, 1973). Salinity near the Bordeaux North point bar is about 1 g L^−1^ (Sottolichio et al., 2011). The suspended matter is composed of clay minerals (60%), fine quartz grains (<10 μm; 25%), calcite (5%), organic matter (3%), and feldspars (6%). The average suspended clay assemblage is composed of illite (49%), smectite (22%), kaolinite (14%), and chlorite (15%; Jouanneau and Latouche, 1981). In the sandy sediments of the point bar, the clay-fraction reached on average 20% of the total sediment volume, with about 7% of smectite, 9% of illite, 2% of chlorite, and 2% of kaolinite (Virolle et al., 2021). At low tide, the exposed part of the point bar can be divided into three zones (Figure 1), from the channel bank to the estuarine point bar: (i) an intertidal mud flat (Figure 1A), (ii) a muddy chute channel extensively covered by gold-colored diatom biofilms (Virolle et al., 2019a; Duteil et al., 2020), and (iii) a low relief (<1 m) formed by the sandy point bar relative to the surface of the chute channel. The surface of the point bar is covered by tidal sand dunes. The point bar is heterolithic, being composed of sand dunes and muddy tidal rhythmites (Allen, 1991). Details of the point bar stratigraphy can be found in Virolle et al. (2021).

## Materials and methods

### Field site description, sample location, and core sampling

The field campaigns to the Bordeaux North point bar were conducted in June 2019. A 6.23 m-long vertical core (BXN Long Core; taken at 44°53′47.40″N, 0°32′24.80″W) was extracted with the EPOC portable vibro-corer (Bordeaux, France) from the chute channel (Figure 1). To protect sediment structures from deformation and fluidization due to water escape, the core barrel was hammered without any rotation and the piston was maintained with a cable. The core was stored at 4°C until opening.

### Core processing and sampling

Cores were photographed upon opening. Two-dimensional *X*-ray images of the cores were obtained with a SCOPIX device (EPOC laboratory, Bordeaux; Migeon et al., 1998), allowing for identification of sedimentary fabrics with a non-destructive approach at high resolution (125 μm). Sixteen 20 cm-long samples (10 cm above and below the target depth) were taken along the core. Samples were homogenized and a subsample was weighed before and after drying in the oven (24 h at 30°C), in order to determine sediment wet and dry weights. A second subsample was kept for measurements of grain size and TOC content, and for cryofixation. The remaining part of the sample was used for EPS extraction, purification, and characterization.

Biofilms were collected by scraping from the surface of the intertidal muddy area (N 44°53′39.10″, W 0°32′22.70″) in the zone of drilling for comparison with core samples (Figure 1A). Surface samples were then split into two portions that were analyzed separately: (i) EPSs extracted from the mud-poor supernatant were called water EPSs and (ii) EPSs extracted from the mud-rich sediment were referred to as mud EPSs.

### Sediment properties

Grain size was measured with a Mastersizer 2000 laser granulometer (Malvern, United Kingdom), with an obscuration rate of 2–25% at the EPOC laboratory (Bordeaux, France). The 16 sub-samples (approximately 1 g) were first sieved to remove large particles (>1,200 μm). Each sample was then measured in triplicate. The median grain size (D_50_, in μm) and the proportion of sand-, silt-, and clay-sized particles were calculated for each sample.

Total organic carbon analyses of sediments were performed with a TOC-LCSH analyzer (Shimadzu, Japan) at the IC2MP laboratory (Poitiers, France) using the Non-Purgeable Organic Carbon method on the 16 core samples. This method consists in adding 2 M HCl to the sample, and then removing dissolved inorganic carbon by air bubbling (2 min 30 s, flow rate: 150 ml.min^−1^, purified air 5.0 quality). Then 50 μl of sample was placed in a furnace at 720°C for complete catalytic combustion and the CO_2_ formed was detected by Infra-Red absorption. TOC concentrations of the liquid samples are expressed relative to a calibration curve established with sodium hydrogenophtalate standard solutions (range 0.2 to 10 mg-CL^−1^). Analyses were performed in duplicate.

### Cryo-scanning electron microscopy

For cryofixation, small fractions of each of the ten samples (nine core samples plus one sample from the surface biofilm) were placed in gold or silver cups (0.9–1 mm in diameter; 0.3 mm deep) and cryofixated under high-pressure freezing (HPF; pressure: 2050 bars) with an EM HPM 100 device (Leica; Switzerland) at the Bordeaux Imaging Center (BIC; Bordeaux, France). Cryofixation prevents the collapse of EPS, allowing optimal observations of three-dimensional organo-mineral relationships. HPF is rapid (freezing occurs within milliseconds) and ensures the simultaneous immobilization of all the EPS macromolecular components. Samples were stored in a cryo-bank (liquid nitrogen; −196°C) until processing. They were subsequently observed under cryo-SEM with a Gemini SEM 300 (Zeiss; Germany) at the BIC. Sublimation, coating, and transfer of the sample to the SEM chamber were done with a cryo-transfer system PP3010T (Quorum Technologies). Mineralogical characterization of the sedimentary particles was performed with an EDX probe at an energy of 12 keV (EDX system: XFlash 6/60; BRUKER, Germany).

### EPS extraction and purification

EPSs were extracted from the core and surface samples by diluting one volume of sample with two volumes of DI water. The solution formed was then put into an ultrasonic bath for 10 min then gently stirred for 4 h. Centrifugation was performed (2,500x*g*, 10 min) to remove the sediments (silt, clay, and sand) and cells. The recovered supernatant was filtered through polycarbonate filters (0.22 μm). The filtrate was precipitated using three volumes of cold propanol (<0°C) per volume of filtrate for a day. The precipitate was recovered by centrifugation (2,800x*g*, 20 min), placed in dialysis tubing (10 kDa), and dialyzed for 3 days in a buffer solution (2 l of DI water at 1 mm EDTA) and three more days against 2 l of DI water (>18 MΩ). Liquid EPS solutions were then stored in the refrigerator at 4°C.

### Physicochemical properties of EPS: Wet assays and acid-base titrations

The composition of EPSs was estimated using three colorimetric assays: (i) phenol-sulfuric acid assay (Dubois et al., 1956), (ii) the Alcian Blue assay (Passow and Alldredge, 1995), and (iii) the Pierce-modified Lowry assay (Lowry et al., 1951). The three assays provide an estimate of (i) the amount of sugar monomers in the sample (i.e., the amount of neutral sugars in EPSs); (ii) the amount of cationic dye binding sites (i.e., the amount of negatively charged reactive sites in the EPSs); and (iii) the amount of proteins. Triplicates (100 μl) were used for each assay. The standards were xanthan for the phenol-sulfuric and Alcian Blue assays, and bovine serum albumin for the Pierce-modified Lowry assay. Absorptions were measured on a Helios Epsilon spectrophotometer (Thermo scientific; cat: 9423UVE100CE).

Acid–base titrations were performed on two EPS samples (surface water EPSs and 6.08 m depth) to determine the potential types and densities of the functional groups present in the EPSs. For that, 3 ml of EPSs were acidified with 100 μl HCL (1 M), and 100 μl of KCL (1 M) were added to provide sufficient ionic concentration. Once acidified, the solution was left under constant nitrogen flow for 20 min to avoid potential CO_2_ dissolution and carbonate or bicarbonate formation during the titration. The solution was titrated with NaOH (0.1 M) added stepwise in 10 μl increments and 5 μl increments when the pH rise was fast. The pH was recorded up to 11 with a pH microelectrode (Sentix 60, A005106A030) connected to a Consort C561 meter (De Bruyne, Belgium). Control titrations were effected with DI water under the same experimental conditions. The titration curves were analyzed using PROTOFIT 2.1 software (Turner and Fein, 2006).

### Fourier transform infrared spectroscopy

Fourier Transform Infrared Spectroscopy (FT-IR) analyses of the 18 EPS samples (16 core samples and 2 surface samples) were performed on a Nicolet IS50 (ThermoScientific) Spectrometer in Attenuated Total Reflection (ATR) mode at the IC2MP laboratory (Poitiers, France). One drop of EPS solution was placed on the diamond ATR element and evaporated. Spectra were acquired between 650 cm^−1^ and 4,000 cm^−1^, with a 4 cm^−1^ spectral resolution and 100 scans were co-added for each spectrum.

### Measurements of microbial activity

The triphenyltetrazolium chloride (TTC) assay was used as a proxy for monitoring metabolically active cells in the sediment. The total reductase activity was measured in 17 samples (in triplicates) through the reduction in triphenyltetrazolium chloride (TTC) to triphenylformazan to characterize metabolic active cells (Relexans, 1996; Braissant et al., 2020). Two grams of sediment sample were homogenized and mixed with 2 ml of 0.8% TTC in Instant Ocean (pH 7.1) to obtain a final concentration of 0.4% TTC. The samples were incubated for 3 h at 30°C. Following the incubation, the samples were centrifuged (3,000 *g*, 10 min) and the pellet was resuspended in 4 ml of acetone to extract the triphenylformazan. After 5 min, the sample was centrifuged (3,000 *g*, 3 min) and the absorbance of the supernatant at 490 nm was measured with a spectrophotometer (Helios Epsilon, Thermo Fisher Scientific). The concentration of formazan was calculated with the Beer–Lambert law with a molar absorption coefficient of 14,320 l.mol^−1^cm^−1^. Blanks were prepared by adding 2 ml of 1.5% glutaraldehyde to the sediment.

The fluoresceine diacetate (FDA) assay was used as a proxy for monitoring the enzymatic activity in the sediment. The hydrolytic activity of the ubiquitous lipase, protease, and esterase enzymes (non-specific hydrolases) was measured in 17 samples (in triplicates) by the hydrolysis of the fluorescein diacetate (FDA) into fluorescein (Green et al., 2006; Braissant et al., 2020). For this, 2 grams of sediment were homogenized in 4 ml of Instant Ocean (pH 7.1) and 60 μl of FDA solution (1 mg.mL^−1^ in acetone). The samples were incubated for 1 day at 30°C. Following incubation, the fluorescein was extracted by adding 4 ml of acetone. After 5 min, the sample was centrifuged (3,000 *g*, 3 min) and the absorbance of the supernatant at 490 nm was measured with a spectrophotometer. The concentration of fluorescein was calculated with the Beer–Lambert law considering a molar absorption coefficient of 73,350 l.mol^−1^cm^−1^. Blanks were prepared by adding 2 ml of 1.5% glutaraldehyde to the sediment. Active diatom cells were used as positive control.

### Statistical analyses

Statistical analyses were performed with the Matlab® R2018b statistic toolbox software. Normality of data was assessed by the Kolmogorov–Smirnov test. As nonparametric distributions were observed, the relationships between EPSs and sediment properties were assessed using Spearman rank correlation as well as principal components analysis (PCA).

## Results

### Vertical evolution of sedimentary facies and sediment properties

**Figure 2:**
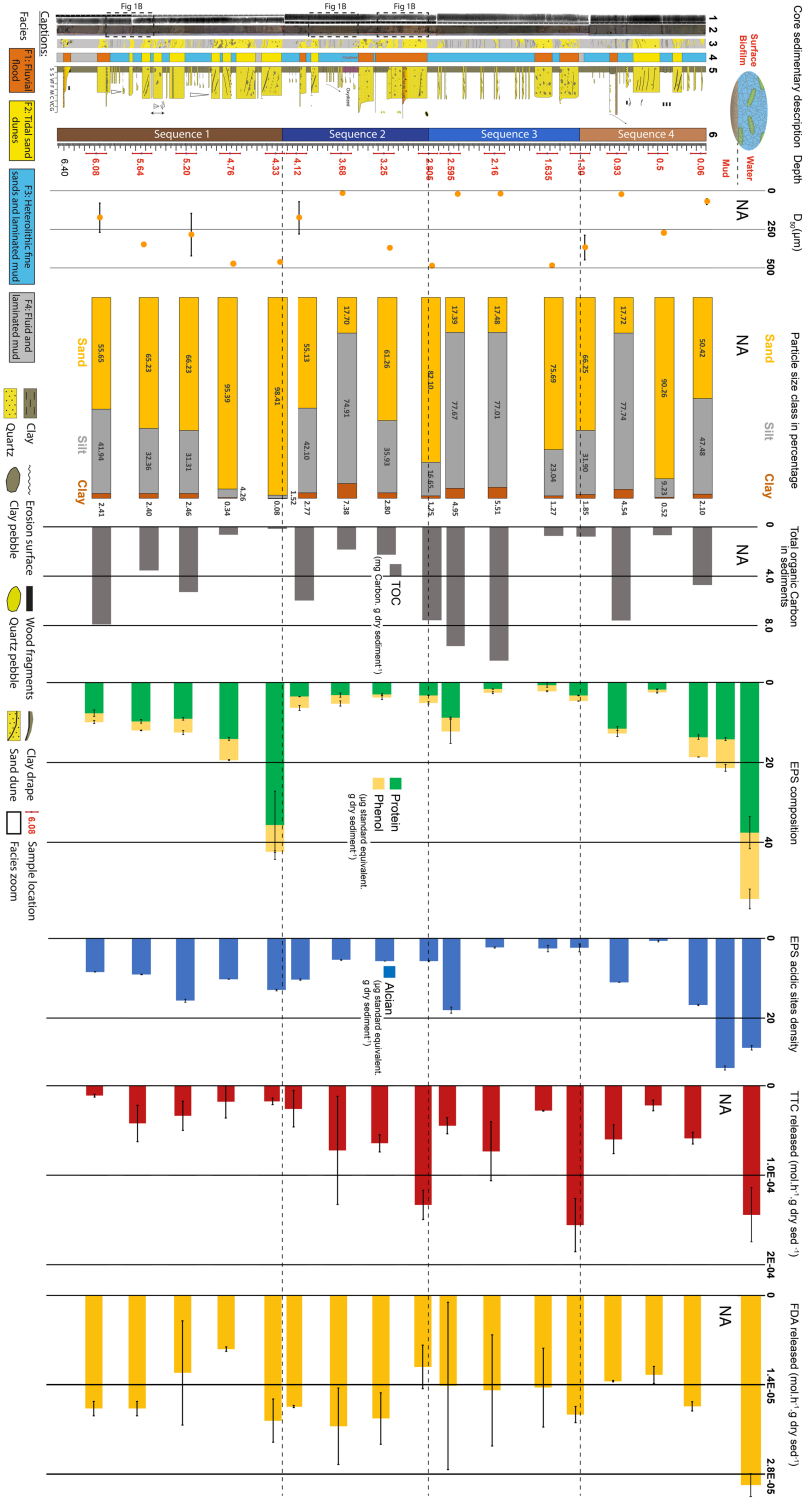
Evolution of exopolymeric substances (EPSs) and sediment properties with depth along the 6 m-long BXN Long Core (see location Figure 1). First column to the left: sedimentary description. (1) *X*-Ray image (SCOPIX); (2) core photographs; (3) sketch; (4) facies; (5) sedimentary log, and (6) sedimentary sequences. Sample depths are written in red. Second column: median grain size (D_50_; yellow dots). Third column: particle size class percentage: clay (brown), silt (gray), and sand (yellow). Fourth column: Sugar and protein concentrations: phenol-sulfuric assay (yellow) and protein assay (green). Fifth column: EPS acidic site density measured with the Alcian Blue assay (blue). Sixth column: total organic carbon (TOC) in the sediment (black). Seventh column: rate of metabolic activity using TTC reduction as a proxy (red). Height column: rate of enzymatic activity using hydrolyzation of FDA as a proxy (yellow).

Four facies were defined in the BXN Long Core (Figure 1B):

Facies 1 (F1; fluvial flood facies) consisted of centimeter- to decimeter-thick horizons of coarse-grained sand mixed with detrital gravels, generally exhibiting a fining-upward trend. These deposits were intercalated with floating centimetric- to multi-centimetric-sized rounded to sub-angular soft mud clasts, some armored with quartz grains (Figures 1, 2). Mud clasts were more abundant at the base, and were locally totally amalgamated. Facies 1 could also be recorded locally as a single erosive surface marked by sparse granule pavements or local accumulation of soft mud clasts over a few centimeters (e.g., between 0.9 and 1 m depth; Figures 1, 2).

Facies 2 (F2; tidal sand dunes facies) was composed of well- to moderately sorted fine-to-medium sands, organized in 10- to 25 cm-thick beds of coarse sands with trough cross-bedding and reactivation surfaces. Dune foresets were locally draped by muds forming mud couplets and were mostly oriented seaward indicating a predominant ebb current (Figures 1, 2).

Facies 3 (F3; heterolithic fine sands and laminated mud facies) was made up of decimeter to meter-thick laminated heterolithic intervals composed of gray to brown laminated clay (lamination was more clearly visible in ScopiX images; Figures 1, 2) and well- to moderately sorted fine-to-medium micaceous sands with occasional centimetric current ripples draped by muds.

Facies 4 (F4; fluid and laminated mud facies) consisted of centimeter to decimeter-thick fluid mud layers alternating with millimeter-thick layers of very fine-grained sands to silty lenses and slack-water clay drapes.

Figure 2 shows the vertical evolution of sedimentary facies, median grain size (D_50_), and the proportion of particle class sizes. The core is dominated by silt (23–78%) and sand (17–90%), while clay-sized particles account for 0 to 7% of the sediment. Four sequences (Sequence 1 to Sequence 4) could be defined based on sedimentary facies, grain size, and bed thickness trends:

- *Sequence 1 (depth 6.46 m to 4.25 m).* The base of sequence 1 (6.46–5.95 m) was dominated by fluid mud (F4). Three sand and mud pebble-rich flood deposits (F1) were found, respectively, at 6.4 m, 6.10 m, and 6.00 m. The top of sequence 1 (Figure 2) was made up of decimeter-thick sand dunes (F2) alternating with fine-grained silty heterolithic layers exhibiting centimeter-thick current ripples (F3). Tidal dunes showed a thickening-upward trend, and the proportion of sand increased from 55 to 98% between 6.08 and 4.33 m. The D_50_ followed approximately the same trend, increasing from 174 ± 93 to 473 ± 7 μm. This sequence was tide-dominated, had in average a mean grain size of 183 μm and was composed in average of 56% sand, 42% silt and 2% clay.
- *Sequence 2 (depth 4.25 m to 2.80 m).* The base of sequence 2 (4.25 m to 3.50 m) was dominated by silty heterolithic layers displaying centimeter-thick current ripples (F3). From 3.50 to 3.65 m, the mud showed a reddish stain. A decimeter-thick fluvial flood layer rich in mud pebbles (F1) was found at 4.05 m and a sandy layer with ebb-oriented dune foresets was found at 3.90 m. The top of sequence 2 (3.50 m to 2.80 m) consisted of successive fluvial flood deposits rich in sand and mud pebbles and bounded by erosive bases (F1). The proportion of sand and the D_50_ increased, respectively, from 61 to 82% and from 371 ± 3 to 487 ± 3 μm in the same depth interval. The uppermost fluvial flood layer was 25 cm thick. This sequence was dominated by river flood deposits had in average a mean grain size of 348 μm and was composed in average of 76% sand, 22% silt and 2% clay.
- *Sequence 3 (depth 2.80 m to 1.30 m).* The base of sequence 3 (1.75 m to 2.80 m) is made up of fine-grained heterolithic layers with current ripples (F3). The proportion of sand reached its lowest relative content (17%) at 2.59 m, while D_50_ reached 20 ± 0.7 μm at 2.16 m. Two decimeter-thick fluvial flood layers with sand and mud pebbles (F1) were found at the top of the sequence (1.70 m and 1.40 m). The proportion of sand and D_50_ increased, respectively, to 75% and 485 ± 5 μm at 1.63 m, and decreased to 66% and 367 ± 79 μm at 1.3 m. This sequence was dominated by river flood deposits had in average a mean grain size of 187 μm and was composed in average of 45% sand, 51% silt and 4% clay.
- *Sequence 4 (depth 1.30 m to the sediment surface)*. The base of sequence 4 consisted in a 5 cm-thick homogeneous fluid layer (F4), overlain by fine-grained heterolithic deposits (F3) from 1.25 to 0.75 m. Two river flood events (F1) were recorded at 1.00 m and 0.93 m as centimeter-thick coarse sand to granules and mud pebble layers with erosive bases. An ebb-oriented sand dune (F2) was found between 0.75 and 0.5 m. The top of sequence 2 was dominated by silty heterolithic deposits (F3). A second ebb-oriented sand dune (F2) was observed between 0.25 and 0.35 m. The proportion of sand increased from 17 to 90% between 0.93 and 0.5 m, and decreased to 50% at 0.06 m. The median grain size follows approximately the same trends, increasing from 24 ± 0.4 to 274 ± 2 μm, then decreasing to 69 ± 19 μm. This sequence was tide-dominated had in average a mean grain size of 183 μm and was composed in average of 56% sand, 42% silt and 2% clay.

TOC fluctuated between 0.17 and 10.84 milligrams of carbon per gram of dry sediment (mg g^−1^ DW). TOC decreased from 7.92 to 0.17 mg g^−1^ DW between 6.08 m and 4.33 m, which was followed by an increase to 5.97 mg g^−1^ DW at 4.12 m. It subsequently dropped to 1.83 mg g^−1^ DW at 3.68 m, and then progressively increased and reached a maximum of 10.84 mg g^−1^ DW at 2.16 m. In the upper part of the core, TOC was below 0.8 mg g^−1^ DW at 1.63 m, 1.30 m, and 0.5 m, and exhibited high values of 7.58 mg g^−1^ DW and 4.72 mg g^−1^ DW at 0.93 m and 0.06 m, respectively.

### Microscopic characterization of biofilms, EPS, and sediments

**Figure 3:**
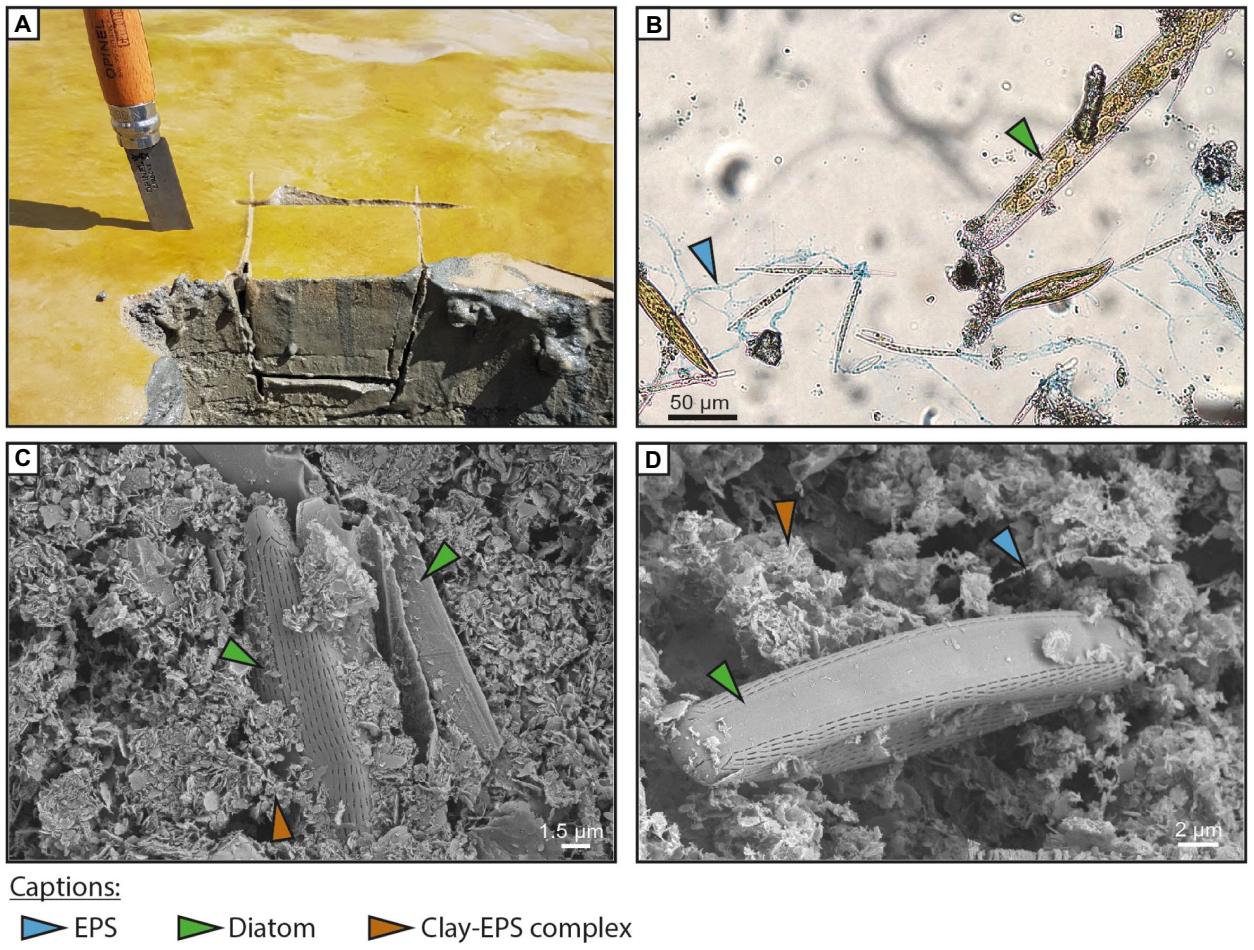
Field and microscope observations of the surface biofilm. Exopolymeric substances (blue arrow), diatoms (green arrow), and clay-EPS complexes (brown arrow). **(A)** Field picture of the diatom biofilm at the surface of the sediment. The biofilm forms a millimeter-thick golden lamina at the surface of the sediment. **(B)** Transmitted light microscopic image of the biofilm colored with Alcian Blue. The blue coloration shows acidic EPS fibers surrounding diatoms. Three diatom genera were identified: *Nitzschia* sp., *Pleurosigma* sp., and *Navicula* sp. **(C)** Cryo-SEM picture of the surface biofilm showing two diatoms surrounded by a mixture of clay and EPSs. EPS fibers form an organo-mineral complex with clay platelets. **(D)** Cryo-SEM picture of a diatom. Cryofixation preserves the 3D structure of EPSs and allows the observation of very thin EPS fibers. The clay-EPS complex displays a typical alveolar structure.

The biofilm formed a golden layer, approximately one millimeter thick, at the surface of the sediment (Figure 3A). Microscopic inspection revealed abundant diatom presence (Figure 1B) and also contained a few green microalgae (e.g., *Euglena* sp.). Three main genera of diatoms were identified: *Nitzschia* sp., *Pleurosigma* sp., and *Navicula* sp. (Lange-Bertalot et al., 2017). Exopolymeric substances (EPSs) occurred as a transparent gel around diatoms and/or within and around clay and silt aggregates. Alcian Blue staining showed that the EPSs were acidic (Figure 3B). Cryo-SEM observations revealed the fine alveolar or filamentous texture of EPSs surrounding diatoms covered with clay- to silt-sized particles (Figures 3C,D).

**Figure 4:**
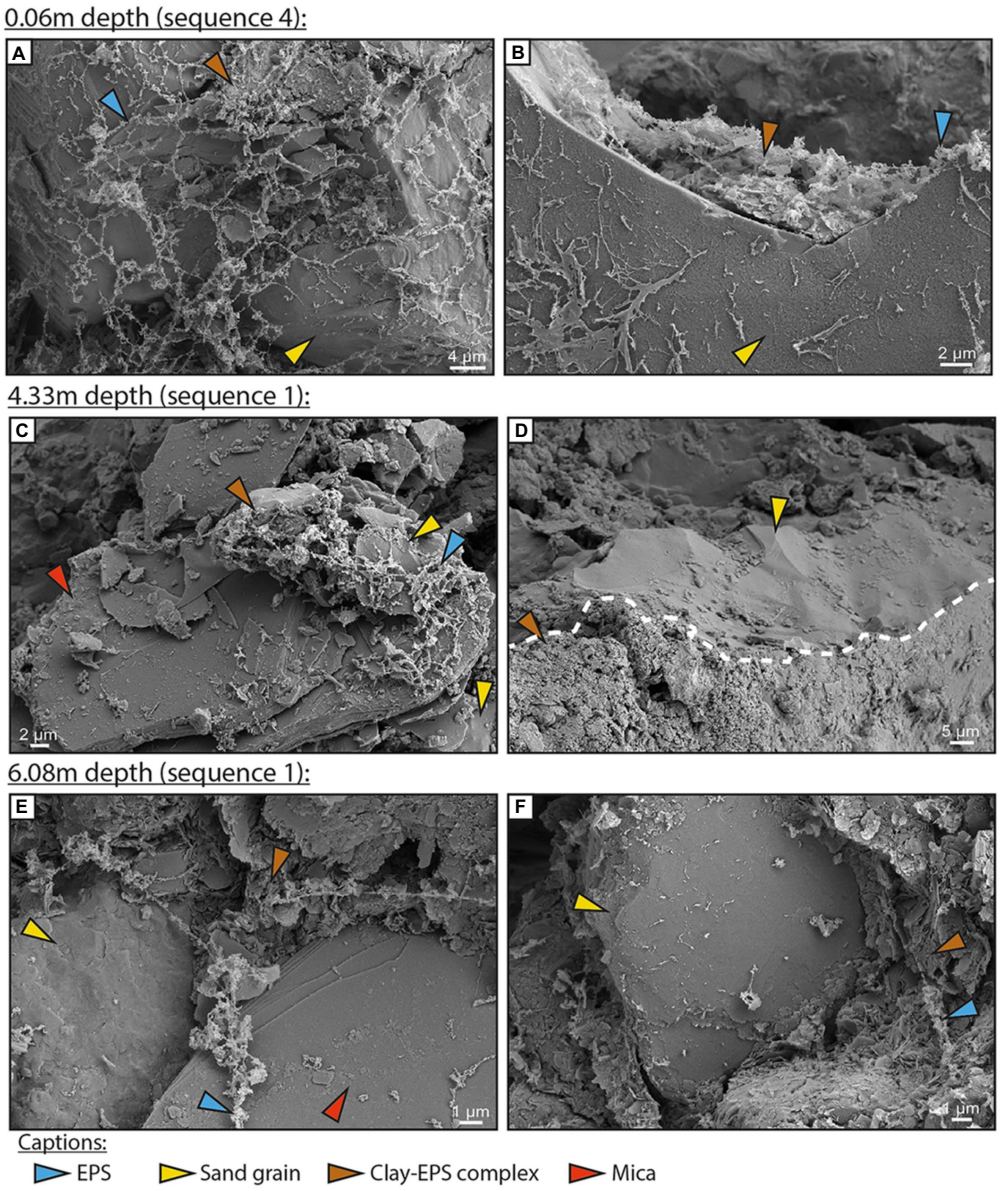
Cryo-SEM images of subsurface sediments from the BXN Long Core at three depths. Exopolymeric substances (blue arrow), quartz grain (yellow arrow), mica (red arrow), and clay-exopolymeric substance (EPS) complexes (brown arrow). **(A,B)** Depth of 0.06 m. Alveolar EPSs cover a large part of a quartz grain. EPSs are locally complexed to clay particles, forming a detrital clay coat. **(C,D)** Depth of 4.33 m. A clay-quartz-EPS complex covers a large part of a mica **(C)**. A sand quartz grain is fully covered by a clay-rich detrital coat **(D)**. **(E,F)** Depth of 6.08 m. EPS fibers are partially covered with clay platelets and coat both quartz and mica grains **(E)**. The surface of a quartz sand grain is partially coated by a dense clay-EPS complex **(F)**.

Diatoms were scarce below the surface biofilm (Figure 4), but EPSs were found at all sampled depths within the core (Figures 2, 4; Supplementary Figures S1, S2). Sand and large silt grains were covered by EPSs alone (Figures 4A,B), or by a detrital coat composed of clay to fine silt attached to EPSs (Figures 4C,F). Similar coats were observed in all sand-dominated layers (4.76, 4.33, 2.80, and 0.50 m). In mud-rich layers, EPSs formed an alveolar network between clay and silt particles (Supplementary Figure S1). The resulting organo-mineral complex was observed in all muddy layers sampled (Figure 4; Supplementary Figures S1, S2). Two examples of EDX profile are available in Supplementary Figures S3, S4.

### Wet chemical assays: Protein, phenol-sulfuric, and alcian blue

The amount of EPSs measured with the three colorimetric assays (i.e., protein, phenol-sulfuric, and Alcian Blue) exhibited large variations with depth (Figure 2; Supplementary Figure S5). For comparison, the results of the assays are expressed as micrograms of EPSs per gram of dry sediment (μg g^−1^ DW), relative to albumin (protein assay) or xanthan (phenol-sulfuric and Alcian Blue assays) standards. The biofilm at the surface of the sediment had the highest EPSs value for the phenol-sulfuric assay (23 ± 3 μg g^−1^ DW), and high values for the protein and Alcian Blue assays (37 ± 5 μg g^−1^ DW and 27 ± 1 μg g^−1^ DW, respectively). The EPSs extracted from the surface mud showed a more than a twofold decrease in EPS amount (14 ± 0.3 μg g^−1^ DW). They also had the highest acidic site concentration (32 ± 0.5 μg g^−1^ DW) but small amounts of proteins (7 ± 1 μg g^−1^ DW). At 0.06 m, the amount of EPSs neutral sugars showed a slight decrease as well as proteins and acidity (4.9 ± 0.05 μg g^−1^ DW, 13.6 ± 0.5 μg g^−1^ DW, and 16.6 ± 0.1 μg g^−1^ DW, respectively). This was followed at 0.5 m by a seven-fold decrease for the phenol-sulfuric and protein assays and by a 24-fold decrease for the Alcian Blue assay. EPS concentrations increased at 0.93 m to 1.2 ± 0.7 μg g^−1^ DW for neutral sugars, 11 ± 0.5 μg g^−1^ DW for proteins and 11 ± 0.05 μg g^−1^ DW for the acidic sites. Between 1.3 and 2.16 m, EPS amounts were low, below 1 μg g^−1^ DW for the neutral sugars, 3 μg g^−1^ DW for proteins and 2 μg g^−1^ DW for acidic sites. At 2.59 m, EPS concentrations increased to 3 ± 3 μg g^−1^ DW for neutral sugars, 8 ± 0.2 μg g^−1^ DW for proteins and 18 ± 0.7 μg g^−1^ DW for acidic sites. Between 2.8 and 4.12 m, EPS concentrations were below 2 μg g DW^−1^ for the phenol-sulfuric assay, 3 μg g^−1^ DW for the protein assay, and 5 μg g^−1^ DW for the Alcian Blue assay. At 4.33 m, concentrations were high, reaching 6.6 ± 0.2 μg g^−1^ DW for neutral sugars, 35.7 ± 9 μg g^−1^ DW for protein, and 13 ± 0.24 μg g^−1^ DW for acidic sites. Between 4.33 m and 6.08 m, EPS concentrations decreased progressively to around 2.2 μg g^−1^ DW for the phenol-sulfuric assay, 8 μg g^−1^ DW for the protein assay, and 8.4 μg g^−1^ DW for the Alcian Blue assay (Table S1).

### Fourier-transform-infrared spectroscopy

**Figure 5:**
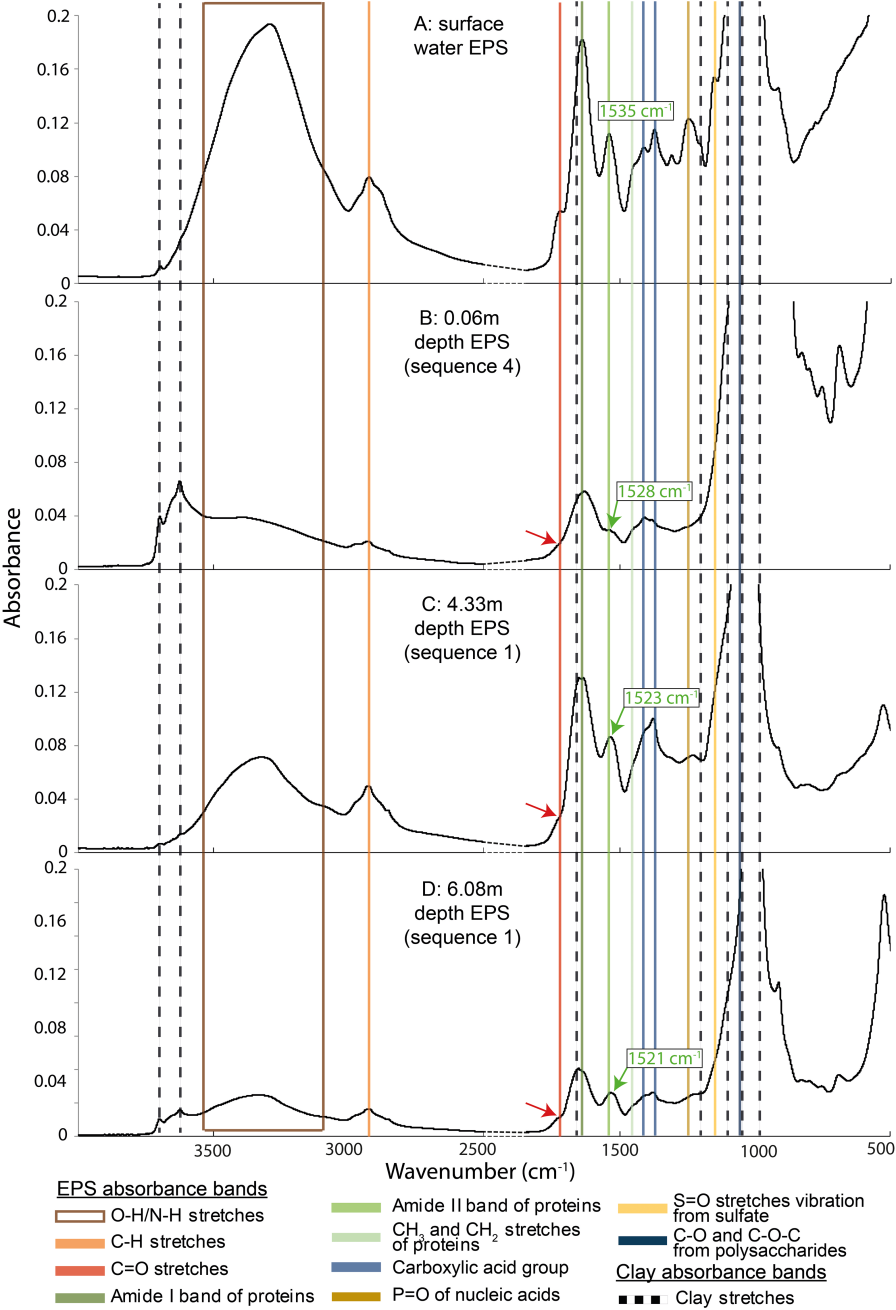
Fourier transform infrared spectroscopy spectra of four exopolymeric substances samples recovered from different depths along the BXN Long Core. Colored lines highlight the EPS absorption bands, while black dotted lines indicate clay mineral absorption bands. The red arrows show the position of the protonated carboxylic acid group stretches, which attenuate or disappear at a certain depth. Green arrows show the shift of the amide II stretches.

EPSs extracted from the core displayed several major infrared absorption peaks typical of polysaccharides and proteins, and also of nucleic acids and lipids (Braissant et al., 2007, 2009; Duteil et al., 2020; Figure 5; Supplementary Figures S6–S8): (i) the vibrational bands in the spectral range 2,800–3,000 cm^−1^ represent the symmetric and antisymmetric stretching of C-H from CH_2_ and CH_3_ groups; (ii) the peak at 1710 cm^−1^ could be attributed to protonated carboxylic acid groups (iii) the peak at 1630 cm^−1^ was likely due to amide I C=O stretching; (iv) the peak at 1535 cm^−1^ could be attributed to the amide II C-N stretching and NH bending; (v) the peak at 1450 cm^−1^ represented the CH_2_ scissoring vibration; and (vi) the doublet at 1404 and 1,365 cm^−1^ was likely due to C-OO^−^ stretching; (vii) the peaks at 1235 cm^−1^ could be attributed to stretching of P=O bond in phosphate and the C-O-C group in esters, and at 1155 cm^−1^ to S=O stretches vibration from sulfate; (viii) the peak at 1024 cm^−1^ represented the carbohydrate C-O stretching vibrations. While present in the surface biofilm EPS, protonated carboxylic acid groups disappeared at 0.06 m and 4.33 m. This group could be characterized again by a very small peak at 6.08 m (Figure 5). The observed frequency variation of amide I between samples (1,619–1,651 cm^−1^) can be explained by the angular deformation of water from EPS or smectite (Chukanov and Chervonnyi, 2016). The amide II band exhibited a shift with depth between the surface and the bottom of the core (from 1535 to 1521 cm^−1^).

While absent from the surface biofilm, clay stretches (Figure 5) of various heights were identified in the 0.06 m sample and below. They could be attributed to one or more of the four clay mineral species found in the estuary, i.e., chlorite, smectite, kaolinite, and illite (Virolle et al., 2019a; Duteil et al., 2020). The four minerals have common around 3,620 (structural OH stretching) and 1,020 cm^−1^ (SiO stretching) that were identified in all samples. Chlorite presents specific peaks at 3550, 3400, and 990 cm^−1^, smectite at 3,400 cm^−1^, and kaolinite at 3,627, 1,113, and 914 cm^−1^ (Bergaya and Lagaly, 2013; Chukanov and Chervonnyi, 2016). Some of these peaks (e.g., Si-O at 1,020 cm^−1^) masked EPS peaks, e.g., carbohydrates C-O at 1,024 cm^−1^. The broad peak between 3,500 and 2,800 cm^−1^ were assigned to water H_2_O stretching vibrations.

### EPS acid–base titrations

**Figure 6:**
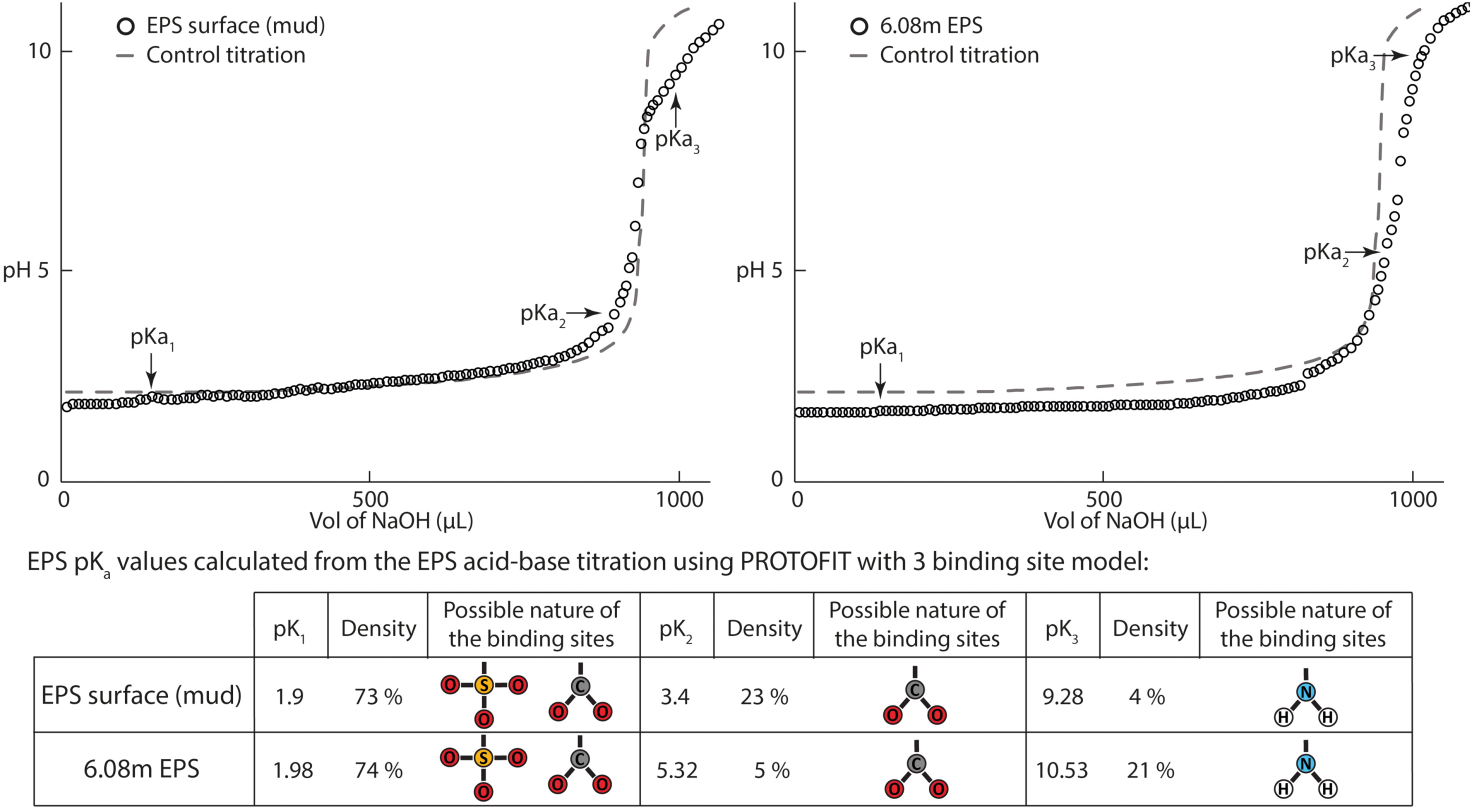
Acid–base titration curves from exopolymeric substances (EPSs) extracted from the surface biofilm and the bottom of the BXN Long Core (depth = 6.08 m). The titration curves obtained were analyzed using PROTOFIT 2.1 software, and both show three buffering zones. The first zone located around pH 2 is attributed to carboxyl or sulfate groups, the second zone located near pH 5 is attributed to carboxyl groups, and the third buffering zone near pH 9.5 is attributed to amino groups. Control titration was performed using the same volume of deionized water adjusted to the same initial pH with HCl 1 M.

Acid–base titrations performed on EPSs extracted from the surface biofilm (water EPSs) and from the bottom of the core (6.08 m depth) exhibited similar patterns and could be described using a model assuming three main buffering zones (Figure 6). These buffering zones correspond to the capacity of the EPSs to counteract the pH changes induced by the addition of NaOH through a release of protons. The first buffering capacity was encountered at a pH close to 2 and could be attributed to carboxyl or sulfate groups. The second buffering zone, found at pH 3.4 in the surface EPSs and at pH 5.52 for the 6.03 m EPSs, could be attributed to carboxyl groups. The third buffering area observed around pH 10 in both samples could be attributed to amine groups. Typical pK values range below 2.8 for sulfates, between 1 and 5 for carboxylic acids, and between 8.5 and 12.5 for amines (Stumm and Morgan, 1996; Braissant et al., 2007). Using Protofit 2.1 software (Turner and Fein, 2006), and assuming three non-electrostatic binding sites, the pKa of the proton-binding sites as well as their densities could be estimated (Figure 6). A first pKa (pK1) between 1.9 and 1.98 was attributed to either carboxyl or sulfate groups and accounted, respectively, for 73 and 72% of site density in the surface and 6.08 m EPSs. The second buffering zone (pK2) was located at 3.4 and represented 23% of site density in surface EPSs. In 6.08 m EPSs, pK2 was found at 5.32, accounting only for 5% of site density. The third buffering zone (pK3) located at 9.28 (surface EPSs) and 10.53 (6.08 m EPSs) was attributed to amino groups, with a higher site density at 6.08 m (21%) than at the surface (4%). These results showed that EPSs predominantly included low pKa sites in both surface and deep sediment EPSs. However, a higher amino group density indicated a potential for deep sediment EPSs to deprotonate at higher pH.

### Microbial activity

The TTC reduction rate were high in the surface biofilm with a rate of 1.44.10^−4^ mol h^−1^ g sed^−1^ DW, and decreased to 2.19.10^−5^ mol h^−1^ g sed^−1^ DW at 50 cm (Figure 2). It subsequently increased to 1.56.10^−4^ mol h^−1^ g sed^−1^ DW between 50 and 130 cm and was low again at 163 cm, with a value of 2.77.10–5 mol h^−1^ g sed^−1^ DW. TTC reduction slightly increased to 7.32.10^−5^ mol h^−1^ g sed^−1^ DW at 216 cm before decreasing to 4.46.10^−5^ mol h^−1^ g sed^−1^ DW at 259 cm and to 1.33.10^−4^ mol h^−1^ g sed^−1^ DW at 2.8 m. The TTC reduction rate then stabilized around 6.5.10^−5^ mol h^−1^ g sed^−1^ DW between 3.25 and 3.68 m and decreased again to 1.79.10^−5^ at 4.76 m. The metabolic activity subsequently increased to 4.2.10^−5^ mol h^−1^ g sed^−1^ DW at 5.64 m depth, before reaching a minimum of 1.1.10^−5^ mol h^−1^ g sed^−1^ DW at 6.08 m.

The FDA hydrolysis rate was relatively high in the surface biofilm, reaching 2.98.10^−5^ mol h^−1^ g sed^−1^ DW, and subsequently decreased to 1.34.10^−5^ mol h^−1^ g sed^−1^ DW at 93 cm (Figure 2). The FDA hydrolysis rate increased to 1.87.10^−5^ mol h^−1^ g sed^−1^ DW at 130 cm, remained relatively stable at approximately 1.4.10^−5^ mol h^−1^ g sed^−1^ DW between 163 cm and 259 cm, before dropping to 1.12.10^−5^ mol h^−1^ g sed^−1^ DW at 280 cm. The enzymatic activity increased and remained constant around 1.95.10^−5^ mol h^−1^ g sed^−1^ DW between 325 cm and 433 cm, decreased to a minimum of 8.43.10^−6^ mol h^−1^ g sed^−1^ DW at 476 cm. The rate of FDA hydrolysis increased to 1.21.10^−5^ mol h^−1^ g sed^−1^ DW at 520 cm, and stabilized to 1.77.10^−5^ mol h^−1^ g sed^−1^ DW between 564 and 608 cm.

### Statistical analyses

**Figure 7:**
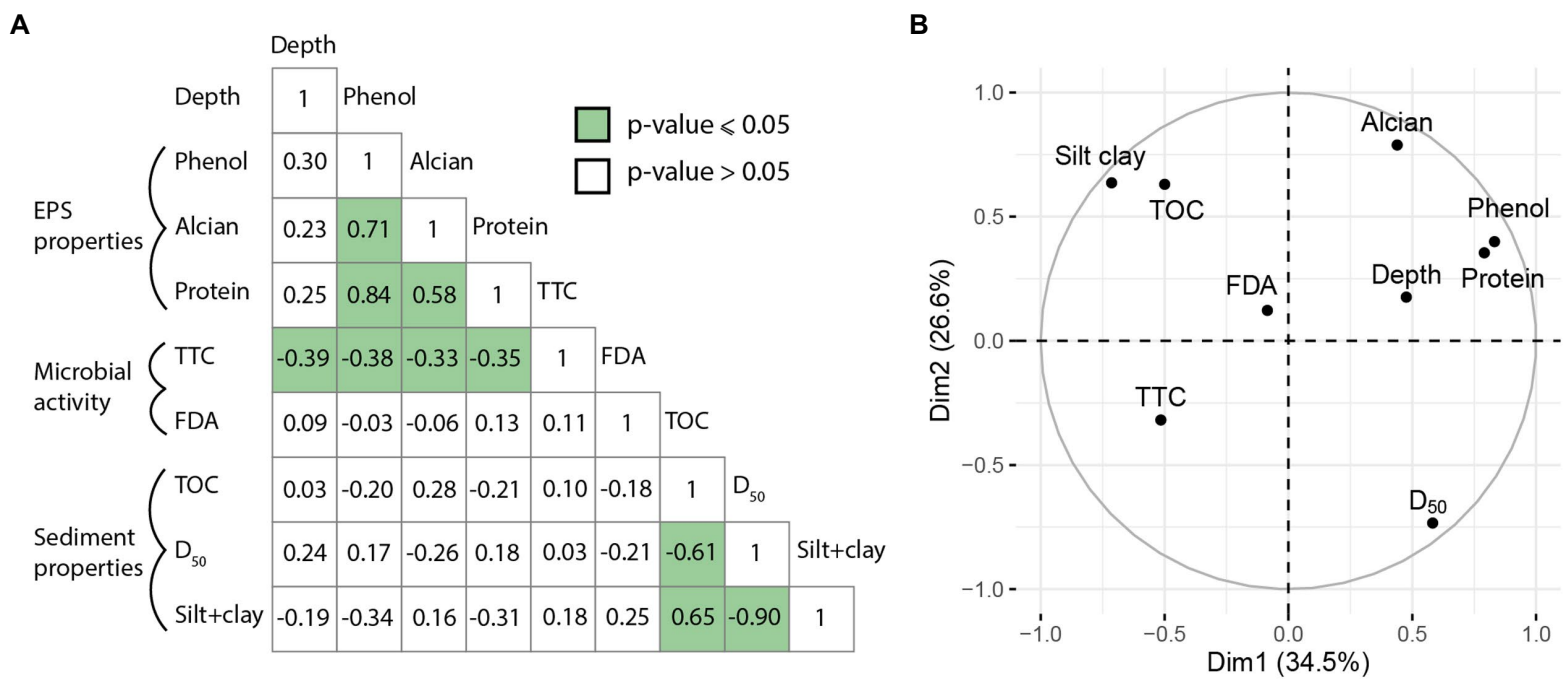
Middle, Principal Components Analysis circle plot of correlation between the samples from the BXN Long Core. **(A)** Correlation matrix of Pearson’s r coefficients between EPS properties, sedimentological factors and depth for the 16 samples. Green boxes indicate value of *p* ≤ 0.05. **(B)** Principal Components Analysis circle plot of variables for BXN-Long Core.

The correlation matrix (Figure 7A) showed significant correlations between sediment grain size and TOC, and between EPS properties (based on three assays). D_50_ and silt+clay were strongly anti-correlated (*n* = 16 samples, value of *p* ≤ 0.05, *r* = − 0.90). TOC was inversely correlated with D_50_ (*n* = 16, *r* = −0.61; value of *p* ≤ 0.05) while being positively correlated with silt+clay (*n* = 16, *r* = 0.65, value of *p* ≤ 0.05), confirming that finer sediments contain more organic carbon (Figure 2). The amount of sugars in EPSs (phenol-sulfuric assay) was positively correlated with the amount of proteins (*n* = 16, *r* = 0.84, value of *p* ≤ 0.05). The Alcian Blue assay (density of acidic sites in EPSs) was positively correlated with both phenol-sulfuric and the protein assays (*n* = 16, *r* = 0.71; value of *p* ≤ 0.05 and *r* = 0.58; value of *p* ≤ 0.05, respectively). The TTC assay was anti-correlated with depth (*n* = 16, *r* = −0.39, value of *p* ≤ 0.05), and with both phenol (*n* = 16, *r* = −0.38, value of *p* ≤ 0.05), Alcian Blue (*n* = 16, *r* = −0.33, value of *p* ≤ 0.05) and protein (*n* = 16, *r* = −0.35, value of *p* ≤ 0.05) assays. There were no statistical correlations between sediment grain size and EPS abundance/reactivity (value of *p* > 0.05), and depth was not a discriminating factor either (value of *p* > 0.05).

The results of the PCA (Figure 7B) were consistent with the correlation matrix. The first axis of the PCA plot (Figure 7B) explained 34.5% of the variation, and the second axis 26.6%. Phenol and protein assays were grouped on the positive side of axis 1, while the Alcian blue assay was discriminated on the positive side of axis 1 and 2. Both EPS proxies were located at the opposite of the TTC assay. TOC and silt+clay were grouped on the negative side on axis 1 and positive side of axis 2, to the opposite of D_50_. FDA was not a discriminant parameter in the PCA. Scatter plot of correlation between the samples from the BXN Long Core is available in the Supplementary Figure S9.

## Discussion

### EPS constituents, extraction, and quantification

Exopolymeric substances are composed of numerous molecules from gels to fully dissolved states that provide the particular physical properties and critical functions of the biofilm (Decho, 2000; Flemming et al., 2016). The EPS matrix is usually separated into two fractions, the bound EPSs and the colloidal EPSs (Nielsen and Jahn, 1999). Bound EPSs (e.g., capsular polymers, condensed gel) are closely associated with cell surfaces, while colloidal EPSs (e.g., soluble macromolecules, “slimes”) are mainly dissolved and interact more with sediments and/or water (Underwood and Paterson, 2003). The two fractions usually have different properties. The colloidal EPSs were particularly targeted in our study due to their reactivity toward sediments (De Brouwer et al., 2002), including the capacity to form organo-mineral complexes with clay minerals (Duteil et al., 2020). Physical methods, particularly centrifugation, are generally efficient for the extraction of colloidal carbohydrates and proteins (Comte et al., 2006). EPSs can be isolated from cells by centrifugation, a method that minimizes cell lysis and contamination by intracellular material (Klock et al., 2007). Due to the large amount of fine sediments in our samples, we added a short ultrasonication step in order to break bonds between EPSs and minerals (Dignac et al., 1998). Furthermore, ultrasonic treatment increases the EPS yield by 1.17 on average (Comte et al., 2006). Chemical extractions including propanol as a solvent ensure that the extracted EPSs are not contaminated by the intracellular material (Comte et al., 2006). EPS precipitation in cold propanol is particularly effective for extracting polysaccharides and proteins with limited structural modification of these molecules (D’Abzac et al., 2010). This precipitation method mostly selects polymers larger than 100 kD (De Brouwer and Stal, 2001), which are key polymers for the physical and chemical properties of biofilm (Decho and Gutierrez, 2017) or interactions with sediment (De Brouwer et al., 2002; Xu et al., 2020). Based on our protocol, we assume that the EPSs extracted in our study include mostly the colloidal fraction and possibly (a small part of) the bound fraction of EPSs.

The concentrations of sugars in EPSs are commonly measured with the phenol-sulfuric acid assay. In estuarine or intertidal mudflats the colloidal EPS concentration for the surface biofilms ranged from 8–10 μg g^−1^ sediment DW (Paterson et al., 2000; Orvain et al., 2014, respectively) to 3 mg g^−1^ DW (Yallop et al., 2000). In these studies, glucose is used as a standard for the phenol-sulfuric assay. In our work, EPS concentrations were measured using xanthan as a standard and were between 0.7 μg g^−1^ DW in some deep sediment horizons and 17 μg g^−1^ DW in the surface biofilm. Xanthan was chosen because it is a large polysaccharide closely related to the molecules naturally present in EPSs (Chenu and Roberson, 1996; Braissant et al., 2009). Xanthan is composed of five hexoses, i.e., it is five times larger than glucose. Even multiplied by 5, the sugar concentrations measured in this study remain largely below 100 μg g^−1^ DW (in glucose equivalent), which places our results in the low to medium range of EPS concentrations in similar environments. Such EPS concentrations could reflect moderate EPS production in the studied area possibly related to seasonal variations. However, the very high sedimentation rates on the Bordeaux North point bar (up to 4 cm yr^−1^; Virolle et al., 2021) could dilute EPSs both in surface and subsurface sediments.

### Relationships between EPSs and sediment grain size

Estuarine microphytobenthic biofilms grow in the intertidal zone of estuaries, preferentially on muddy and stable substrates (e.g., Underwood and Paterson, 2003; Orvain et al., 2012). However, EPS components show a differential response to sediment grain size. In surface sediments or short sedimentary cores (<30 cm), the amount of colloidal carbohydrates is often positively correlated with the proportion of mud (i.e., particles <63 μm; Paterson et al., 2000; Hope et al., 2020), while proteins or bound carbohydrates are not (Gerbersdorf et al., 2009a). Similar observations were made in laboratory experiments, with greater secretion of colloidal carbohydrates by diatoms on mud than on sand, while bound proteins were more abundant in sand-mud mixtures (Ubertini et al., 2015). Our results show no correlation between grain size, and the amounts of sugars, proteins, or the acidity of EPSs in the 6 m-long core BXN-LC (Figures 2, 7), indicating that grain size does not exert a major influence on EPS preservation in subsurface sediments.

Light attenuation is greater in muddy sediments than in sands. While light penetrates just the uppermost millimeter of muds, it can persist up to several millimeters or centimeters in sands (Kühl et al., 1994; Cartaxana et al., 2011). Distinct assemblages, of epipelic and epipsammic diatoms, develop on mud and sand. Epipelic diatoms are able to migrate vertically through the sediment as a possible adaptation to lower and more fluctuating light conditions, and/or to avoid resuspension (Jesus et al., 2009). A high level of production of EPSs in mud and sand mixtures could reflect a balance between light and nutrient availability (Morelle et al., 2020). Biofilm development could be less efficient at the surface of sands, but persist deeper, attenuating the difference in EPS concentrations between sandy and muddy facies. Besides, erosion of EPSs by tidal currents could be intensified in sands due to their greater permeability (Ubertini et al., 2015). Underwood and Kromkamp (1999) estimated that 30 to 50% of the EPSs produced in intertidal biofilms could be transported during a semi-diurnal cycle. Strong tidal currents and wind waves cause resuspension and redistribution of sediments including EPS-sediment complexes (e.g., Underwood, 2010; Redzuan and Underwood, 2021), which could contribute to the homogenization of EPS concentrations in low-energy muddy and high-energy sandy areas (Duteil et al., 2020).

### Production and consumption of EPSs in the sediment

Our results show that the photosynthetically active diatom biofilm has the highest concentrations in colloidal EPSs (both sugars and proteins) in the intertidal zone of the Gironde estuary (Figures 2, 4). We also show for the first time that significant amounts of EPSs can be preserved several meters deep in estuarine sediments (Figure 4). Benthic diatoms produce EPSs with functions (e.g., motility in the sediment, adhesion to the sediment, protection against desiccation) allowing their establishment and sustainability on the estuary sedimentary bottom, despite highly fluctuating light conditions, hydrodynamics or frequent emersions (Decho, 2000). In sedimentary environments, EPSs are highly concentrated and reactive in the biofilms developing at the surface of the sediment and within the first millimeters of the sediment, owing to photoautotrophic production (Braissant et al., 2009; Pace et al., 2018). The diatom genera present in the Gironde epibenthic biofilms (e.g., *Navicula*, *Nitzschia*) are efficient EPS producers, as observed in cultures (Underwood and Paterson, 2003).

EPS quantities generally decrease with depth, as documented in several sedimentary environments such as river sediments (Gerbersdorf et al., 2009a), estuarine mudflats (Paterson et al., 2000; De Brouwer and Stal, 2001), hypersaline lake microbial mats (Braissant et al., 2009; Pace et al., 2018), and tropical soil crusts (Mager, 2010). The decrease in the amount of EPSs and in their reactivity with depth is interpreted as the result of (i) lowered photoautotrophic EPS production below the photic zone of the biofilm/mat and (ii) increased heterotrophic consumption associated with aerobic and anaerobic respiration (Decho et al., 2005; Braissant et al., 2009; McKew et al., 2013). This is also marked in our results with a strong metabolic and enzymatic activity in the surface sediment (Figure 2). Within the sediment, heterotrophs rapidly (within days) consume the smaller and more labile fraction of the EPSs (also LMWOC), while larger and refractory molecules are degraded more slowly (Decho et al., 2005; Braissant et al., 2009). In intertidal sediments, anaerobes are more efficient than aerobes at degrading the large and refractory EPS molecules produced by diatoms into smaller molecules (i.e., LMWOC; McKew et al., 2013).

In the Gironde estuary, we observe a decrease in the amount and acidity of EPSs near the surface and at a depth of 0.5 m (Figure 2), which was combined with a disappearance of the carboxylic acid peak in FT-IR from 0.06 m (Figure 5; Supplementary Figures S4, S5). This decrease in EPS concentrations and acidity coincides with a decrease in metabolic and enzymatic activities (Figure 2). Diatoms are known to produce sulfate-rich polysaccharides (Bhaskar et al., 2005). The presence of sulfate groups in the Gironde water EPS (Figure 5) could indicate a greater contribution of diatoms to the surface EPS pool. The disappearance of the sulfate peak in deeper horizons (Figure 5) could result from the consumption of these sulfate-rich EPSs by heterotrophs (Battersby et al., 1984; Visscher et al., 1999). The consumption of low-molecular-weight organic compounds (LMWOC) reduces the number of acidic functional groups in EPSs (Braissant et al., 2009). Such a phenomenon could explain the decreasing EPS concentration and reactivity observed from the surface biofilm to a depth of 0.5 m, but also the low values observed between 1.30 and 4.12 m (except for 2.59 m). In this interval, some horizons (e.g., 1.30 m and 2.80 m) show a significant metabolic activity, as indicated by elevated rates of TTC reduction (Figure 2). By contrast, some deeper horizons contained EPSs in comparable amounts to the surface biofilm (e.g., 4.33 m; Figure 2). The relatively high acidic sites densities observed at 0.93 m, 2.59 m, 4.33 m, or 5.20 m (Figure 2) could indicate a relatively low degree of EPS degradation in these horizons, especially since the metabolic activity is low and the enzymatic activity is moderate (Figure 2).

To our knowledge, this is the first report of reactive EPSs several meters deep in sediment. These EPSs could be remnants of former diatom biofilms. The degradation of photosynthetically produced EPSs by heterotrophs may result in the formation of more refractory EPS compounds (Decho et al., 2005). Some extracellular proteins produced by bacteria or diatoms, such as amyloid fibrils, are also highly resistant to heterotrophic degradation (Decho and Gutierrez, 2017), which could explain the high protein concentrations in some deep horizons from the studied core (e.g., 0.93 m, 4.33 m, 4.76 m; Figure 2).

Aerobic and anaerobic heterotrophs inhabiting sediments also secrete EPSs, including abundant extracellular enzymes (Marvasi et al., 2010; Decho and Gutierrez, 2017). For instance, sulfate-reducing bacteria (SRB) produce EPSs rich in polysaccharides in culture and probably within hypersaline microbial mats (Braissant et al., 2007). Being abundant in estuarine sediments (Leloup et al., 2005), SRB could be significant contributors to the subsurface (or endogenous) EPS pool of the Gironde estuary. Other anaerobes such as methanogens are known to produce polysaccharide and protein-rich EPSs (Veiga et al., 1997), and are also present within estuarine sediments (McKew et al., 2013). The differentiation between low degradation rates of EPSs and high degradation coupled to high heterotrophic production rates (high turnover) in the various sediment horizons is not possible with our data. This could be achieved for example by using tracers such as ^14^C-labeled EPSs, and by measuring their conversion into ^14^CO_2_ (Decho et al., 2005).

### EPS-mineral interactions and EPS preservation

Exopolymeric substances play a major role in the interactions between sediments and microorganisms, although the ecological functions of EPSs differ between biofilm-forming groups. In intertidal biofilms, most benthic diatoms secrete EPSs for motility on the sediment in response to semi-diurnal cycles, while some bacteria use EPSs to attach to the substratum, i.e., sedimentary particles (Smith and Underwood, 1998; Lubarsky et al., 2010). As a consequence, some EPS molecules exhibit high reactivity toward mineral surfaces (Dontsova and Bigham, 2005; Kleber et al., 2007; Ghashoghchi et al., 2017; Hope et al., 2020). EPSs can complex to mineral surfaces through hydrogen-bonding, van der Waals forces, hydrophobic/hydrophilic bonding, and cation bridges (Kleber et al., 2015; Duteil et al., 2020). In the Gironde estuary, EPS acidity peaked in the mud-rich part of the surface biofilm, indicating a potential strong cation binding capacity (Braissant et al., 2009; Pace et al., 2018). Acid–base titrations and FT-IR show that the same EPSs included functional groups that could interact with mineral surfaces, e.g., carboxylic acids, sulfate and amino groups, and also hydrophobic parts of proteins (Figures 5, 6; Duteil et al., 2020). For example, carboxylic acid groups may interact with the hydroxyl groups or the metal cations on the edge of the clay minerals through hydrogen-bonding (Kleber et al., 2007). The proportion of functional groups varied through the studied core (Figures 2, 5, 6). The contribution of amino groups is of 4% in the surface biofilm (Figure 6), but increased to 21% at 6.08 m (21%; Figure 6). In contrast, the contribution of carboxylic acid and/or sulfate groups was more pronounced at the top than at the bottom of the core (Figure 6). Given the range of porewater pH measured in the core (7.9–8.3; unpublished data), carboxyl and sulfate groups could be deprotonated at all sediment depths and available for interactions with metallic cations and with clay mineral surfaces through cation bridging (Duteil et al., 2020).

In the surface biofilm, EPSs formed aggregates together with clay and silt-sized particles, mainly clay minerals, micas, and quartz (Figures 3B,D). Similar aggregates were observed at all depths in the core (Figure 4). In sand-rich horizons, EPSs or EPS-clay aggregates covered large parts of the surface of sand grains (quartz or micas), forming detrital clay coats. Clay-coated grains account for 30% of sands in the point bar (Virolle et al., 2021), for 17 to 25% of sands in the tidal bars from the bay-head delta in the same estuary (Virolle et al., 2019b), and up to 50 to 90% of sands in the Ravenglass estuary (UK; Wooldridge et al., 2017; Worden et al., 2020). Wooldridge et al. (2017) reported a positive correlation between carbohydrate concentrations and the proportion of clay-coated grains in the sediment, which was not the case in the present study. Detrital clay coats were formed experimentally at ambient surface conditions by mixing purified diatom biofilm EPSs with quartz and clay minerals, confirming a prominent role of EPSs in the aggregation of mud and sand (Duteil et al., 2020). Natural diatom biofilms include numerous satellite bacteria, and experiments have shown that mixed diatom-bacteria assemblages produce EPSs with higher cohesive properties than axenic diatom cultures or pure prokaryotic cultures (Lubarsky et al., 2010). The EPSs secreted by anaerobic heterotrophic bacteria also promote clay aggregation. Jaisi et al. (2007) demonstrated experimentally that the combination of iron (III) reduction and exopolysaccharide production by iron-reducing bacteria induces the aggregation of nontronite. Some molecules of the EPSs could also be integrated within the interlayer space of dioctahedral smectites (Duteil et al., 2020), which represent an average of 7% of the mineralogical assemblage in the point bar studied by Virolle et al. (2021). Interactions of proteins and polysaccharides with minerals result in significantly stronger adhesion of organisms constituting the biofilm and in increased biofilm and sediment stability (Gerbersdorf et al., 2009b; Flemming and Wingender, 2010). An increased binding between EPS moieties and mineral surfaces could also reduce the efficiency of EPSs extraction, leading to an underestimation of EPS concentrations in some sedimentary horizons. Based on the absence of correlation between EPSs and both sediment grain size and depth, we estimate that this effect is probably very low. Chemical extraction techniques (e.g., EDTA) could help increase EPS yields. However, we voluntarily avoided this type of approach that can induce contamination of the samples (Comte et al., 2006).

**Figure 8:**
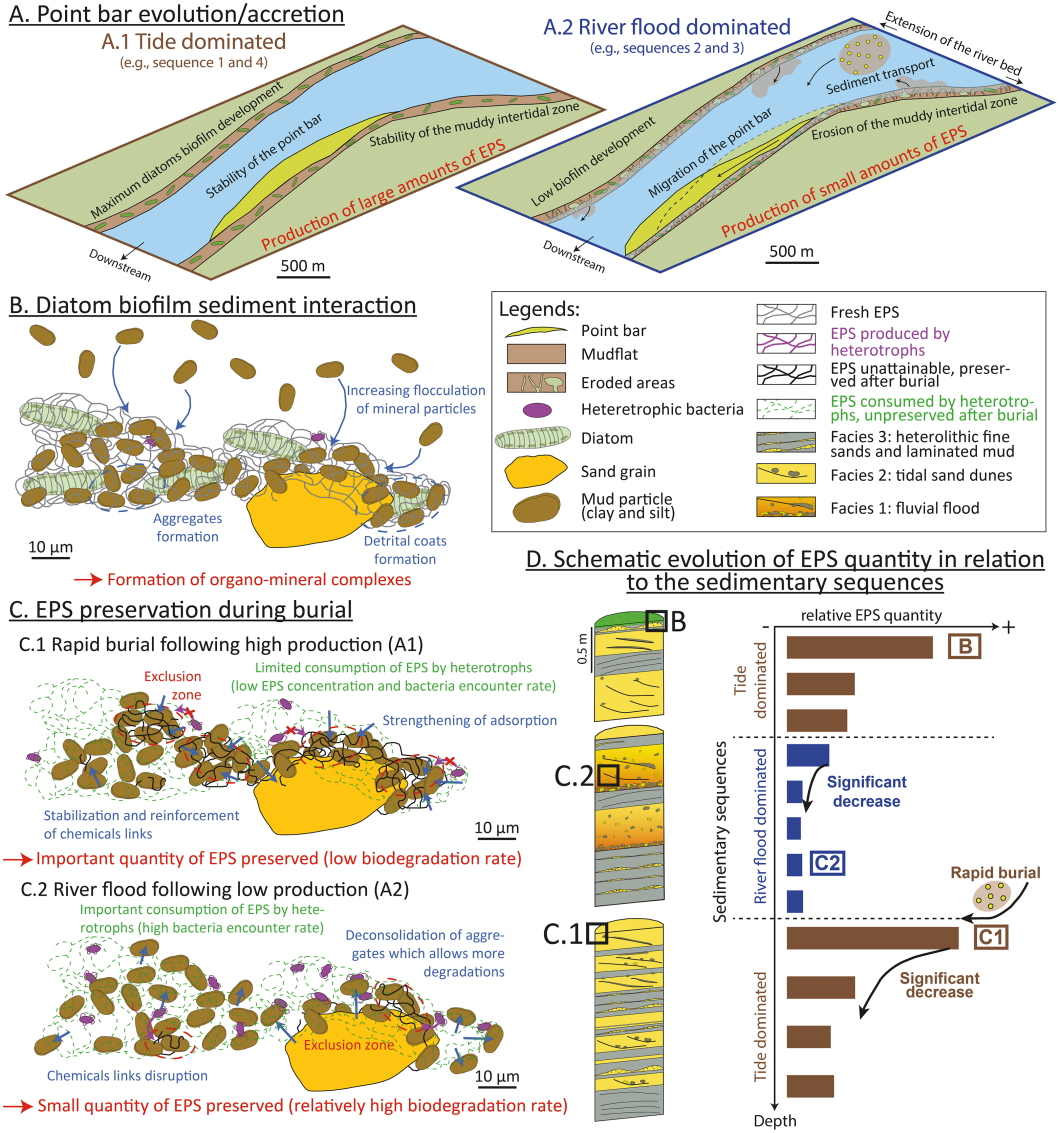
Multiscale scenario explaining the role of the hydro-sedimentary context and organo-mineral interactions in the production and preservation of exopolymeric substances (EPSs) in estuaries. **(A)** Sketch of the Bordeaux North point bar during a tide-dominated period (A.1; brown) and a river flood-dominated period (A.2; blue). Biofilm development and EPS production are optimal during tide-dominated intervals, when the point bar is stable and the muddy intertidal domain well developed. During river flood-dominated periods, the migration of the point bar coupled to the erosion of the intertidal zone results in lower EPS production. **(B)** Formation of clay-EPS complexes: clay-EPS aggregates and detrital coats in surface sediments. **(C)** The preservation of clay-EPS complexes and EPSs during burial depends on the sedimentation rate: (C.1) rapid burial following, e.g., a river flood protects EPSs from heterotrophic degradation; and (C.2) normal sedimentation rate leads to increased EPS degradation. **(D)** Vertical trends of EPS concentrations depend on production and sedimentation. Tide-dominated periods followed by rapid burial create the optimum conditions for EPS preservation, while river flood-dominated periods followed by tide-dominated periods are associated with low EPS preservation.

The factors controlling the preservation of organic matter and its subsequent storage in sediments have been largely discussed (e.g., Zonneveld et al., 2010). Two hypothesis could explain EPS preservation in the Gironde subsurface sediment:

- EPSs could persist because their concentration is below a threshold for biological utilization at the microscale, as it is the case for the dissolved organic carbon pool (DOC; Traving et al., 2015). In experiments, Arrieta et al. (2015) demonstrated that low concentrations of DOC were associated with slow microbial growth, because intrinsically labile compounds were at concentrations too low to compensate the metabolic costs associated with their utilization. On the Bordeaux Nord estuarine point bar, the sedimentation rate can reach an average of 4 cm yr^−1^ (Virolle et al., 2021). This high sedimentation rate could dilute EPSs, increasing their preservation by reducing their potential encounter with EPS-consuming heterotrophs within the sediment.
- Clay-EPS aggregation may reduce EPS consumption through an occlusion of the porosity within aggregates, particularly through hydrophobic interactions (Kleber et al., 2015). EPSs extracted from the Gironde sediments contained proteins (Figures 2, 5, 6) including molecules prone to such interactions (Lubarsky et al., 2010). The shift of the amide II peaks (Figure 5) could indicate a structural change in the secondary structure of the proteins upon stronger interactions with mineral surfaces (Dousseau and Pezolet, 1990; Kleber et al., 2007). Aggregates could form exclusion zones with mineral surfaces protecting EPSs against degradation by heterotrophs (Marshman and Marshall, 1981; Kleber et al., 2015; Figure 8). Long-term interactions during sediment burial (over centuries in the present study) may have consolidated the bonds between EPSs and mineral surfaces, initially formed at the surface (Duteil et al., 2020; Figure 8). The reduction in porosity and mechanical reorganization of grains due to sediment compaction probably had very little influence, given the shallow burial of the sediments in the core under study (Rebata-Landa and Santamarina, 2006).

While dissolved EPSs could be easily metabolized by heterotrophs at the surface of sediments, EPSs preservation within sediment could be explained by the combination of two phenomena. EPSs complexation to mineral surfaces could protect them from degradation, while their dilution in the sediment could decrease the probability of their encounter with heterotrophs.

### Potential tide versus river flood control on EPS preservation

Both historic maps of the Gironde estuary and radiometric ages measured in sedimentary cores indicate that the Bordeaux North point bar is less than 300 years old (Virolle et al., 2021). During the last century, the Bordeaux North point bar experienced a rapid lateral accretion rate (1 m yr^−1^ on average), with an average sedimentation rate of between 1 and 4 cm yr^−1^ (Virolle et al., 2021). In the studied core (BXN Long Core), sedimentary sequences 1 and 4 are dominated by muddy (Facies F3) and sandy (Facies F2) tidal facies whereas sequences 2 and 3 exhibit fine-grained tidal facies (Facies F3) overlain by river flood deposits (Facies F1; Figures 1B, 3). Similar vertical successions have been described in tidal bars from the Gironde bay-head delta, *ca.* 30 km downstream from the study area. In the Plassac area of the estuary, thick fine-grained heterolithic layers similar to facies F3 could result from low-water discharge during dry periods, while it is thought that thick sands rich in mud-pebbles similar to facies F1 accumulate during subsequent peak floods (Chaumillon et al., 2013). In the Gironde estuary, the dimensions and migration (e.g., longitudinal vs. lateral accretion) of tidal bars and point bars (including the Bordeaux North point bar) responds to multi-annual to decadal variations of river discharge (Fenies and Tastet, 1998; Billy et al., 2012; Gascuel, 2017). A stratigraphic correlation with a core located *ca.* 100 m south of the investigated area and dated using Cs-Pb (core Bo-2016-W; Virolle et al., 2021) indicates that the uppermost 1.5 m of the BXN Long Core, i.e., sequence 4, could have been deposited during the last seven decades. From the 1950s until the 2010s, the point bar displays relative stability, with a near-constant volume and no major migration (Gascuel, 2017). Over the same period, river discharges have been relatively low compared to the first half of the 20th century (Billy et al., 2012), leading to the turbidity maximum zone (TMZ) migrating several kilometers upstream in the Garonne channel (Jalón-Rojas et al., 2021). Decreasing river discharges could have led to an increase in fine sediment trapping (e.g., Virolle et al., 2019a) and to the deposition of the tide-dominated sediments described in sequence 4 (Figure 2). Frequent and intense river floods can result in significant erosion, especially in the inner parts of estuaries (Luan et al., 2016). River discharge was high from the 1900s to the 1940s, with numerous episodes of river floods (Billy et al., 2012), some of which might be recorded in sequences 2 and 3. Over the same period, the Bordeaux North point bar underwent significant longitudinal accretion (Gascuel, 2017). Based on these observations, the sedimentary succession described in this study (Figure 2) could record successive phases of point bar development alternatively dominated by tides (sequences 1 and 4) and by river floods (sequences 2 and 3). We propose a conceptual model of the sedimentary evolution of the point bar during both types of sequences, and of subsequent biofilm development, EPS production, and EPS preservation (Figure 8).

Tide-dominated sequences show higher average EPS concentrations (4.93 μg xanthan eq g^−1^ DW for neutral sugars; 15.68 μg albumin eq g^−1^ DW for proteins; Figure 2) than river flood-dominated sequences (1.87 μg xanthan eq g^−1^ DW for neutral sugars; 3.35 μg albumin eq g DW^−1^ for proteins; Figure 2). EPS concentrations are very high at the top of tide-dominated sequences, and decrease with depth (Figure 2), while river flood-dominated sequences show less marked vertical trends. EPS acidity is almost twice as high on average in tide-dominated sequences as in river flood-dominated sequences (12.6 vs. 6.67 μg xanthan eq. g DW^−1^, respectively, Figure 2). However, while acidic site density decreases overall with depth in sequence 1, it shows no particular vertical trend in sequence 4 (Figure 8). In river flood-dominated sequences, acidity tends to increase slightly with depth (Figure 2). Except for the surface sediment, the highest metabolic activities are measured in river flood facies (F3; Figure 2).

We propose a scenario describing potential interactions between the sedimentary dynamics of the point bar and the production and preservation of EPSs (Figure 8). However, it should be noted that this model is based on the results from one core, and that additional cores will be required to test the proposed hypotheses. Tide-dominated sequences (sequences 1 and 4) could be characterized by a stable point bar and by a substantial extension of the muddy intertidal zones (Figure 8A.1). The stability of the sediment substrate could favor the development of biofilms, leading to significant photoautotrophic EPS production at the surface. With increasing depth, EPS concentration decreases (Figure 2) due to heterotrophic degradation (Figure 8D). During river flood-dominated sequences (sequences 2 and 3), sustained erosion of the mudflats and migration of the point bar could result in reduced development of intertidal biofilms and in lower EPS surface production (Figure 8A.2).

Sediment-EPS aggregates, including clay coats, form at the surface of the sediment (Virolle et al., 2019a; Duteil et al., 2020; Figure 8B). This aggregation could create exclusion zones protecting EPSs from heterotrophic degradation by preventing bacteria from reaching EPSs (Figure 8C). The preservation of significant amounts of EPSs, as observed at the transition between sequences 1 and 2 (4.33 m deep) could result from the combination of: (i) high EPS production at the top of a tide-dominated sequence, followed by (ii) a rapid burial by river flood deposits, preventing aerobic degradation of EPSs. Rapid burial of surface EPSs could strengthen the chemical links between EPSs and mineral surfaces and promote the formation of stronger and less degradable aggregates (Figure 8C.1). In contrast, river flood-dominated deposits overlain by tide-dominated deposits could result in reduced EPS preservation, as observed at the transition between sequences 3 and 4. Lower initial EPS production followed by increased dismantling of aggregates and EPS degradation due to low sedimentation rates could explain the low EPS concentrations observed in sequences 2 and 3 (Figure 8C.2). A single flood event within a tide-dominated sequence could also carry biofilm material and EPSs from upstream, explaining the slightly higher EPS concentration at 0.93 m (Figure 2). More generally, our results demonstrate a potential strong sedimentary and hydroclimatic control on EPS production and preservation in estuaries. Our proposed model could be tested in other estuaries, as well as in other sedimentary environments.

## Conclusion

Exopolymeric substances (EPSs) are often considered as degraded below the first millimeters of the sediment. In this study, we extracted and characterized EPSs from a 6 m-long core drilled in an estuarine point bar. EPS composition and physicochemical properties were compared to sediment grain size, total organic carbon (TOC), sedimentary facies, depth, and enzymatic and metabolic activities. The core could be subdivided into four sedimentary sequences, S1 to S4 from older to younger. S1 and S4 exhibited a predominance of tidal facies (e.g., tidal sedimentary dunes, flaser bedding), while S2 and S3 displayed abundant river flood facies (e.g., erosive, mudclast-rich facies). The Gironde surface and subsurface EPSs were rich in polysaccharides and proteins, including reactive functional groups, mainly carboxylic acids and amino groups, but also possibly sulfates. Such EPSs were present in variable concentrations down to 6 m deep in the sediment. EPSs were abundant and highly reactive in the surface diatom biofilm, and decreased sharply in the first decimeters of the sediment. However, we measured EPS concentrations comparable to the surface biofilm 4.33 m deep in the sediment. This is the first report of EPSs at such depth. FT-IR spectroscopy and acid–base titrations showed minor changes in the composition of EPSs with depth: (i) a local disappearance of carboxylic acid groups in some horizons, (ii) a modification of amino groups with depth possibly linked to complexation with mineral surfaces, and (iii) an increase in amino groups relative to carboxyl or sulfate groups at depth.

Sedimentary particles (e.g., sand, silt, and clay) and EPSs formed organo-mineral complexes (e.g., detrital clay coats) at the surface of the sediment and these aggregates were preserved at all depths in the core. Such aggregates could preserve EPSs from heterotrophic degradation. The concentration of EPSs was not statistically correlated with sediment depth, sediment grain size or TOC, but was anti-correlated with the metabolic activity measured with the TTC assay. EPS concentrations are higher overall in sedimentary sequences dominated by tidal facies than in sequences dominated by river flood facies. Such sequences could form over several decades. Based on these results, we proposed a scenario including a combination of factors that could explain the presence and preservation of EPSs several meters below the sediment–water interface. EPS production could be maximum at the end of tide-dominated periods, when the estuarine point bar is stable and the muddy intertidal zone is most extensive. In contrast, river flood periods could be associated with significant point bar migration and erosion of the intertidal zones. This instability could prevent significant development of biofilms.

The preservation of EPSs could be favored by the combination of (i) their dissolution in the sediment lowering their potential encounter rate with bacterial cells and (ii) the formation of EPS-sediment aggregates protecting EPSs against heterotrophic degradation. The microenvironment at depth becomes essentially stable inhibiting bacteria contact with substrate. Rapid burial by muddy deposits following a tide-dominated sequence could provide the best conditions for EPS preservation. In contrast, the dismantling of sediment-EPS aggregates during river flood could increase EPSs availability and degradation. A part of the subsurface EPSs could also be produced *in situ* by heterotrophs. In brief, the quantity of EPSs preserved in the sediment could reflect the changes in the sedimentary dynamics of the estuary (i.e., tide vs. river floods) modulating EPSs production and consumption. Highly reactive preserved EPSs could play a major role in early diagenetic organo-mineral reactions.

## Data availability statement

The original contributions presented in the study are included in the article/Supplementary material, further inquiries can be directed to the corresponding author.

## Author contributions

TD, RB, and OB: field, experimental design, laboratory handling, and manuscript writing. BG: FT-IR acquisition and manuscript writing. ML: TOC measurements. EP and HF: sedimentary description and sediment core analysis. BB: sedimentary description and manuscript writing. IS: cryo-SEM and EDX. AH: statistical analysis and field. YY: radiocarbon datation. PV: field, experimental design, and manuscript writing. All authors contributed to the article and approved the submitted version.

## Funding

This study was financed by a doctoral contract from the French Ministry of Research and Higher Education and received funding from the TelluS programme of the French Institute of Earth and Planetary Sciences, CNRS (ELAPSE project, 2019). The work was initiated as part of the CLAYCOAT project “CLAY COATing in Shallow Marine Clastic Deposits to Improve Reservoir Quality Prediction,” a collaborative project between the Université Paris-Saclay, Bordeaux INP, Université Bordeaux Montaigne, Université de Poitiers, and Neptune Energy. PV acknowledges support from I-SITE, grant number UB18016-BGS-IS to the Université de Bourgogne Franche-Comté.

## Conflict of interest

EP was employed by the company 45-8 Energy.

The remaining authors declare that the research was conducted in the absence of any commercial or financial relationships that could be construed as a potential conflict of interest.

## Publisher’s note

All claims expressed in this article are solely those of the authors and do not necessarily represent those of their affiliated organizations, or those of the publisher, the editors and the reviewers. Any product that may be evaluated in this article, or claim that may be made by its manufacturer, is not guaranteed or endorsed by the publisher.

## Supplementary material

SUPPLEMENTARY FIGURE S1 Cryo-SEM images of sediments from the BXN Long Core at three depths. Exopolymeric substances (blue arrow), quartz grain (yellow arrow), mica (red arrow) and clay-EPS complexes (brown arrow). **(A,B)** depth of 0.5 m. Alveolar EPSs cover a large part of a quartz grain **(A)**. EPSs are complexed to clay particles, forming a detrital clay coat, which includes quartz grains **(B)**. **(C,D)** Depth of 0.93 m. A quartz grain is covered by a clay-rich detrital coat **(C)**. A sand quartz grain is embedded in a clay-EPS coat **(D)**. **(E,F)** Depth of 1.63 m. A centric diatom frustule covered with EPS lies on a quartz grain covered by clay and mica **(E)**. The surface of a quartz sand grain is partially coated by a dense clay-EPS complex **(F)**.

SUPPLEMENTARY FIGURE S2 Cryo-SEM images of sediments from the BXN Long Core at three depths. Exopolymeric substances (blue arrow), quartz grain (yellow arrow), mica (red arrow) and clay-EPS complexes (brown arrow). **(A,B)** depth of 2.805 m. Fragment of a diatom frustule within a dense clay-EPS matrix also embedding a mica particle **(A)**. Rounded quartz grain displaying a detrital clay coat **(B)**. **(C,D)** Depth of 3.68 m. A quartz silt is covered by a clay-EPS detrital coat **(C)**. A centric diatom frustule is engulfed in the clay-EPS complex that also traps a silt and mica particles **(D)**. **(E,F)** Depth of 4.76 m. Quartz grain covered by a clay-EPS complex **(E)**. The surface of a quartz sand grain is partially coated by a dense clay-EPS complex **(F)**.

SUPPLEMENTARY FIGURE S3 Cryo-SEM images and corresponding EDX profiles of the 4.33 m depth sample. **(A)** A mica (area 1) is coated by a clay-EPS complex (point 2) containing a quartz silt grain (point 3). **(B)** A quartz grain (point 1) is coated by clay (point 2).

SUPPLEMENTARY FIGURE S4 Cryo-SEM images and corresponding EDX profiles of the 6.08 m depth sample. **(A)** and **(B)** additional pictures displaying mica and quartz grain coated by clay-EPS organo mineral complex. **(C)** A quartz grain (area 2) and a mica (area 1) are stuck in clay-EPS complex (point 3). **(D)** A quartz grain (point 1) is covered with a clay-EPS organo-mineral complex (points 2 and 3).

SUPPLEMENTARY FIGURE S5 Data used in this study: EPS properties, microbial activity and sediment properties.

SUPPLEMENTARY FIGURE S6 FTIR spectra of four exopolymeric substances samples recovered from different depths along the BXN Long Core (0.5, 0.93, 1.30 and 1.635 m depth). Colored lines highlight the EPS absorption bands, while black dotted lines indicate clay mineral absorption bands.

SUPPLEMENTARY FIGURE S7 FTIR spectra of four exopolymeric substances samples recovered from different depths along the BXN Long Core (2.16, 2.595, 2.805 and 3.25 m depth). Colored lines highlight the EPS absorption bands, while black dotted lines indicate clay mineral absorption bands.

SUPPLEMENTARY FIGURE S8 FTIR spectra of four exopolymeric substances samples recovered from different depths along the BXN Long Core (3.68, 4.12, 5.20, 5.64m depth). Colored lines highlight the EPS absorption bands, while black dotted lines indicate clay mineral absorption bands.

SUPPLEMENTARY FIGURE S9 Principal Components Analysis scatter plot of correlation between the samples from the BXN Long Core. Colors highlighted the main facies found in the core, facies 1 in orange, facies 2 in yellow and facies 3 in blue.
