# Supplementary figures and images for: Preservation of exopolymeric substances in estuarine sediments
Source: Front Microbiol. 2022 Aug 18;13:921154. doi: 10.3389/fmicb.2022.921154 (PMC9434125; doi:10.3389/fmicb.2022.921154)

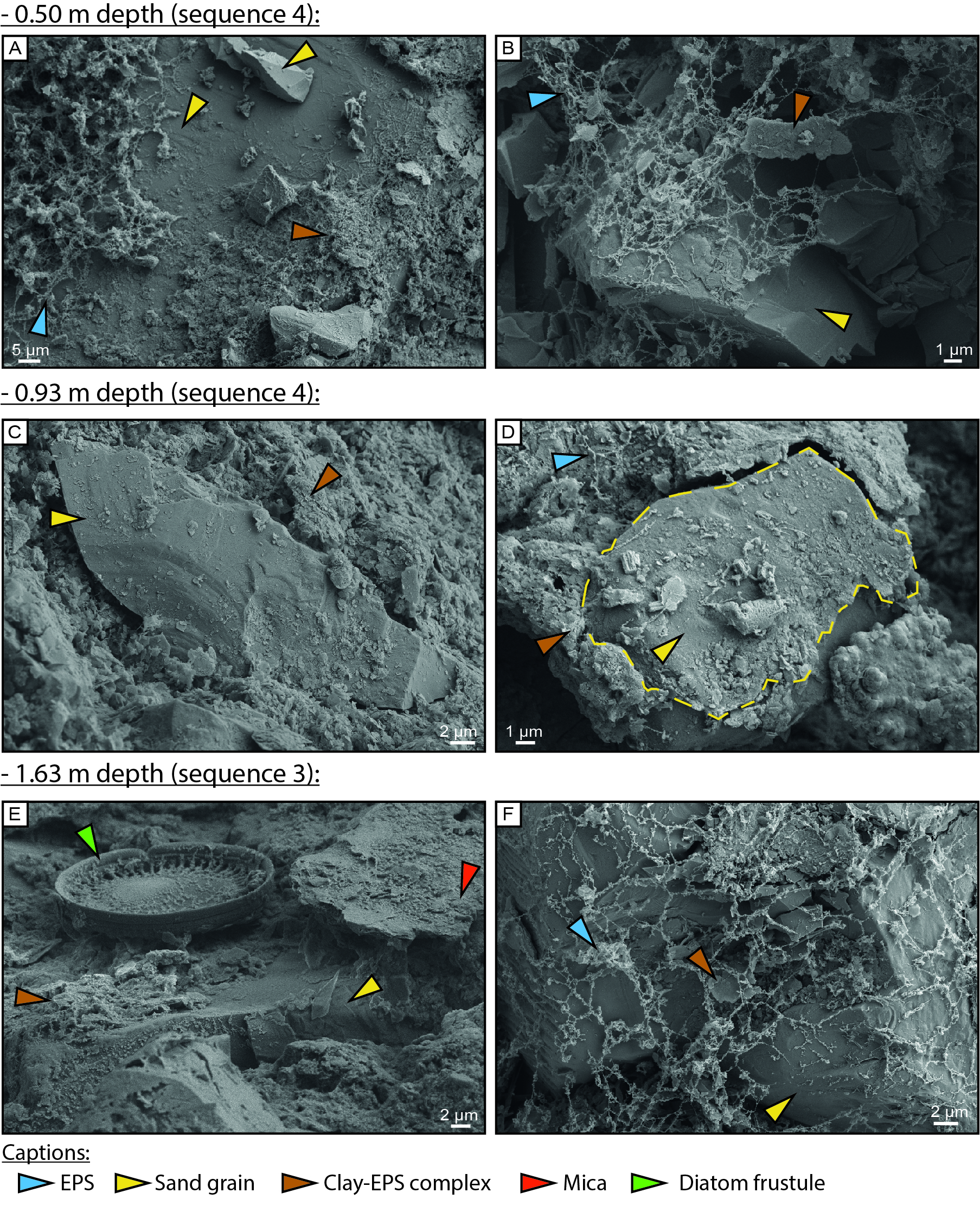

Supplement: SUPPLEMENTARY FIGURE S1 — Cryo-SEM images of sediments from the BXN Long Core at three depths. Exopolymeric substances (blue arrow), quartz grain (yellow arrow), mica (red arrow) and clay-EPS complexes (brown arrow). (A,B) depth of 0.5 m. Alveolar EPSs cover a large part of a quartz grain (A). EPSs are complexed to clay particles, forming a detrital clay coat, which includes quartz grains (B). (C,D) Depth of 0.93 m. A quartz grain is covered by a clay-rich detrital coat (C). A sand quartz grain is embedded in a clay-EPS coat (D). (E,F) Depth of 1.63 m. A centric diatom frustule covered with EPS lies on a quartz grain covered by clay and mica (E). The surface of a quartz sand grain is partially coated by a dense clay-EPS complex (F). [file Image_1.JPEG]

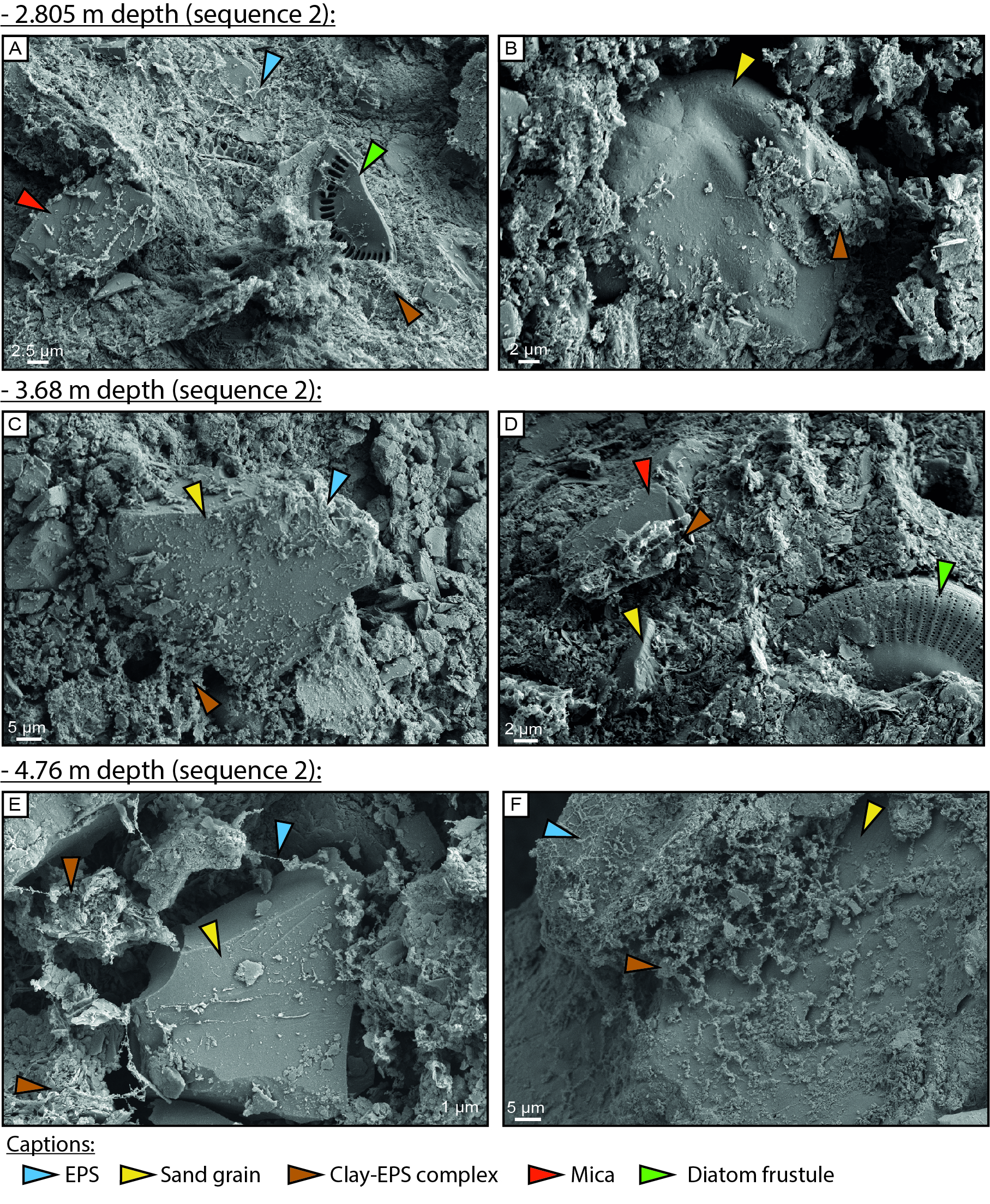

Supplement: SUPPLEMENTARY FIGURE S2 — Cryo-SEM images of sediments from the BXN Long Core at three depths. Exopolymeric substances (blue arrow), quartz grain (yellow arrow), mica (red arrow) and clay-EPS complexes (brown arrow). (A,B) depth of 2.805 m. Fragment of a diatom frustule within a dense clay-EPS matrix also embedding a mica particle (A). Rounded quartz grain displaying a detrital clay coat (B). (C,D) Depth of 3.68 m. A quartz silt is covered by a clay-EPS detrital coat (C). A centric diatom frustule is engulfed in the clay-EPS complex that also traps a silt and mica particles (D). (E,F) Depth of 4.76 m. Quartz grain covered by a clay-EPS complex (E). The surface of a quartz sand grain is partially coated by a dense clay-EPS complex (F). [file Image_2.JPEG]

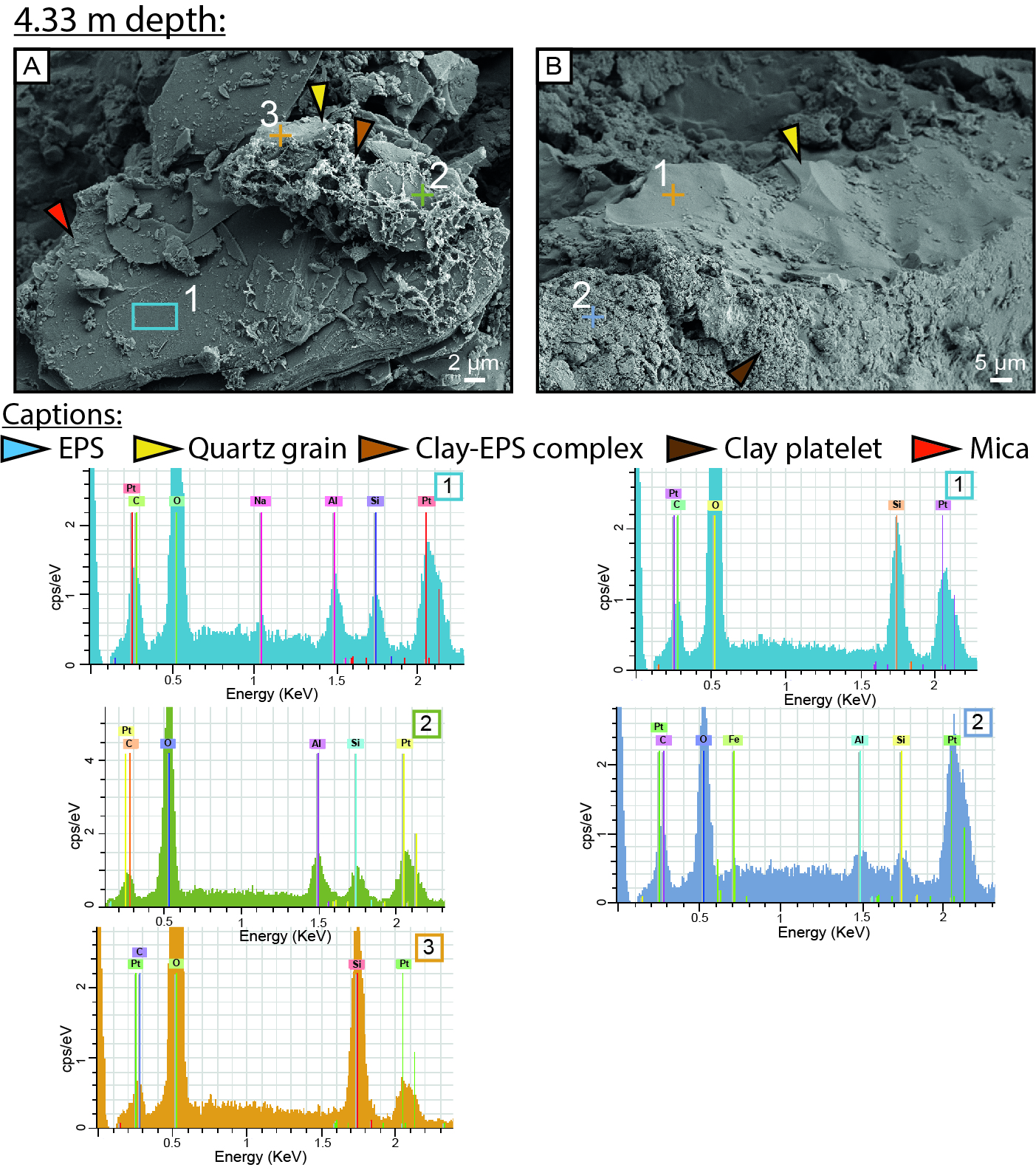

Supplement: SUPPLEMENTARY FIGURE S3 — Cryo-SEM images and corresponding EDX profiles of the 4.33 m depth sample. (A) A mica (area 1) is coated by a clay-EPS complex (point 2) containing a quartz silt grain (point 3). (B) A quartz grain (point 1) is coated by clay (point 2). [file Image_3.JPEG]

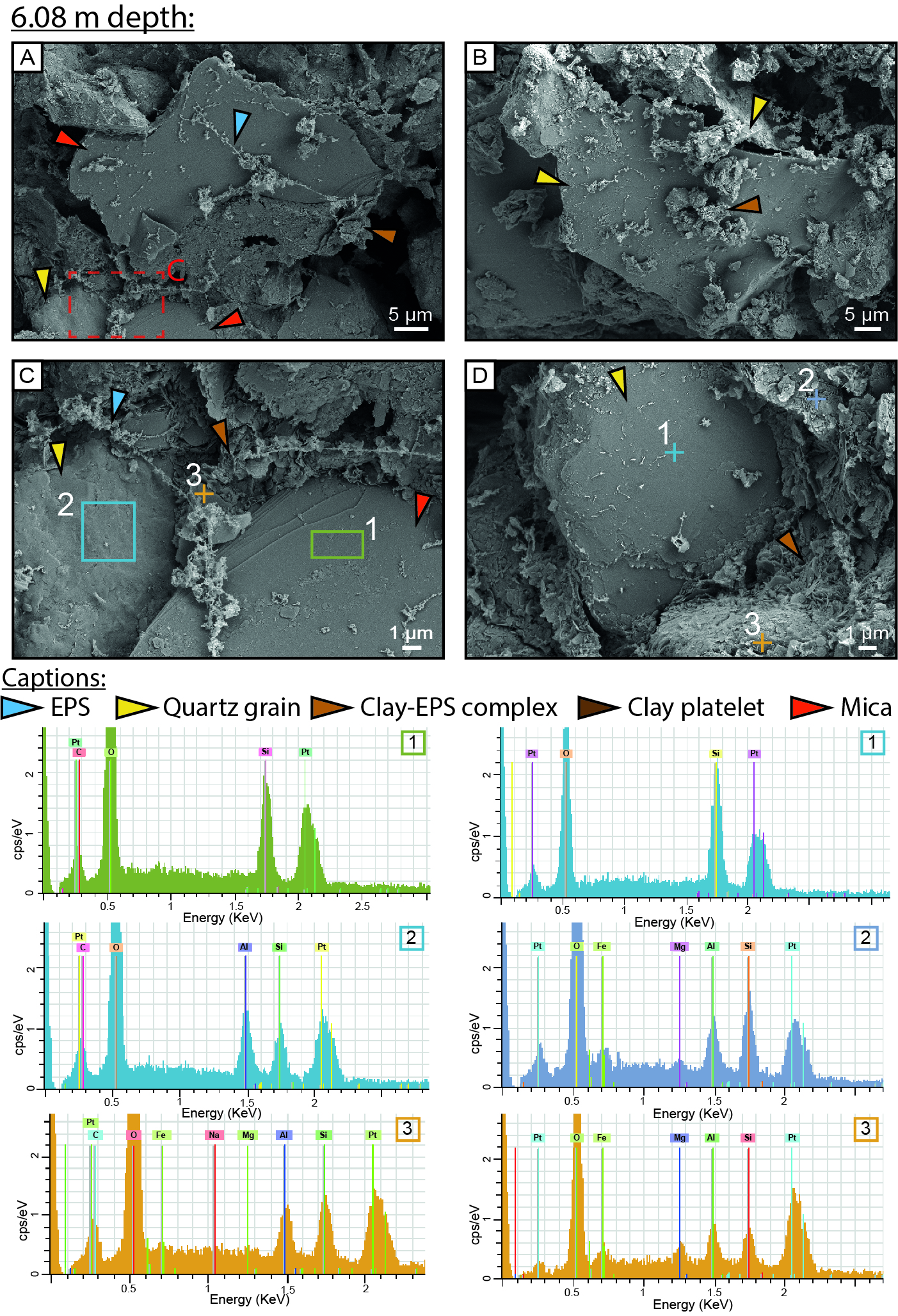

Supplement: SUPPLEMENTARY FIGURE S4 — Cryo-SEM images and corresponding EDX profiles of the 6.08 m depth sample. (A) and (B) additional pictures displaying mica and quartz grain coated by clay-EPS organo mineral complex. (C) A quartz grain (area 2) and a mica (area 1) are stuck in clay-EPS complex (point 3). (D) A quartz grain (point 1) is covered with a clay-EPS organo-mineral complex (points 2 and 3). [file Image_4.JPEG]

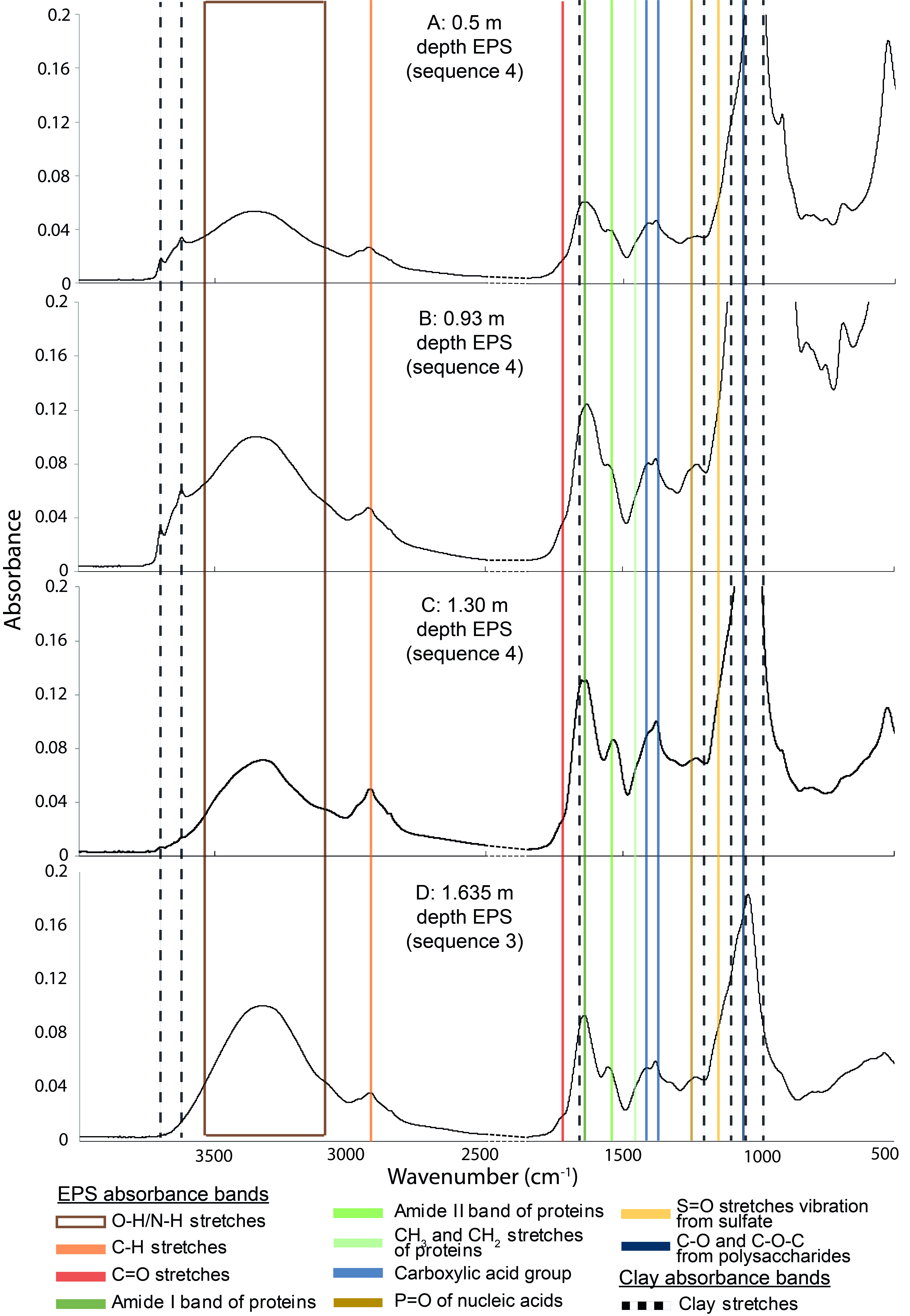

Supplement: SUPPLEMENTARY FIGURE S5 — Data used in this study: EPS properties, microbial activity and sediment properties. [file Image_5.JPEG]

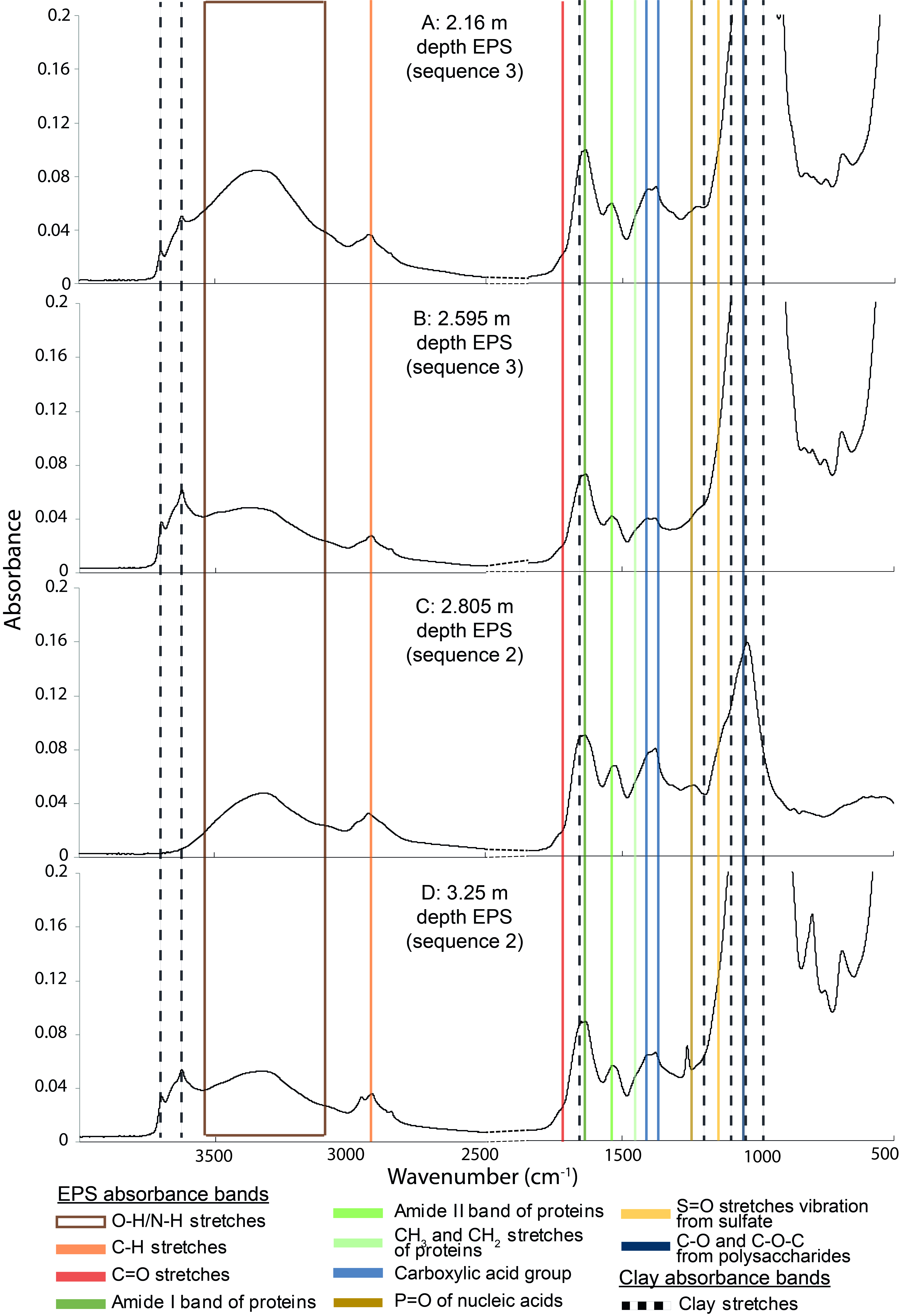

Supplement: SUPPLEMENTARY FIGURE S6 — FTIR spectra of four exopolymeric substances samples recovered from different depths along the BXN Long Core (0.5, 0.93, 1.30 and 1.635 m depth). Colored lines highlight the EPS absorption bands, while black dotted lines indicate clay mineral absorption bands. [file Image_6.JPEG]

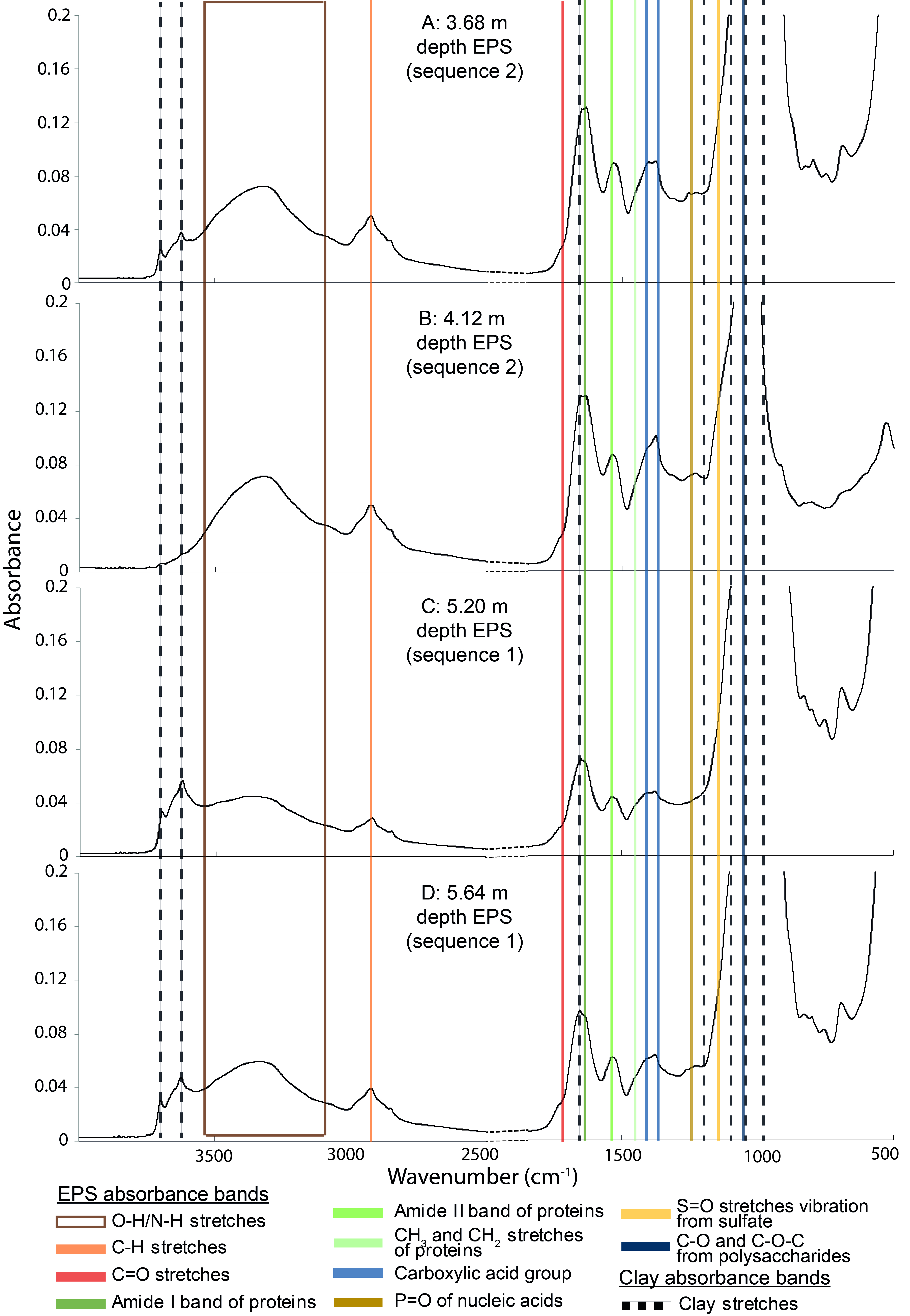

Supplement: SUPPLEMENTARY FIGURE S7 — FTIR spectra of four exopolymeric substances samples recovered from different depths along the BXN Long Core (2.16, 2.595, 2.805 and 3.25 m depth). Colored lines highlight the EPS absorption bands, while black dotted lines indicate clay mineral absorption bands. [file Image_7.JPEG]

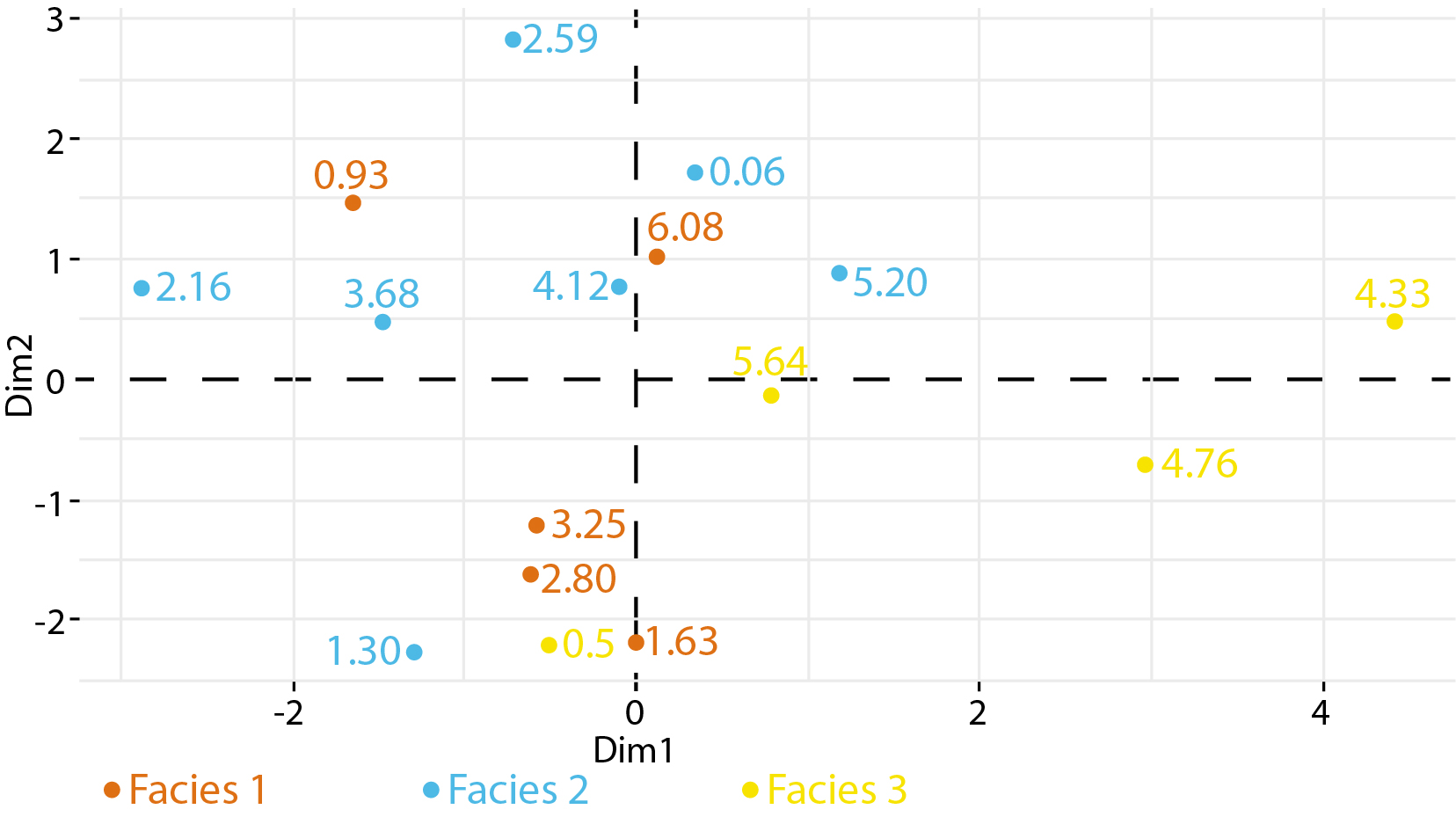

Supplement: SUPPLEMENTARY FIGURE S8 — FTIR spectra of four exopolymeric substances samples recovered from different depths along the BXN Long Core (3.68, 4.12, 5.20, 5.64m depth). Colored lines highlight the EPS absorption bands, while black dotted lines indicate clay mineral absorption bands. [file Image_8.JPEG]

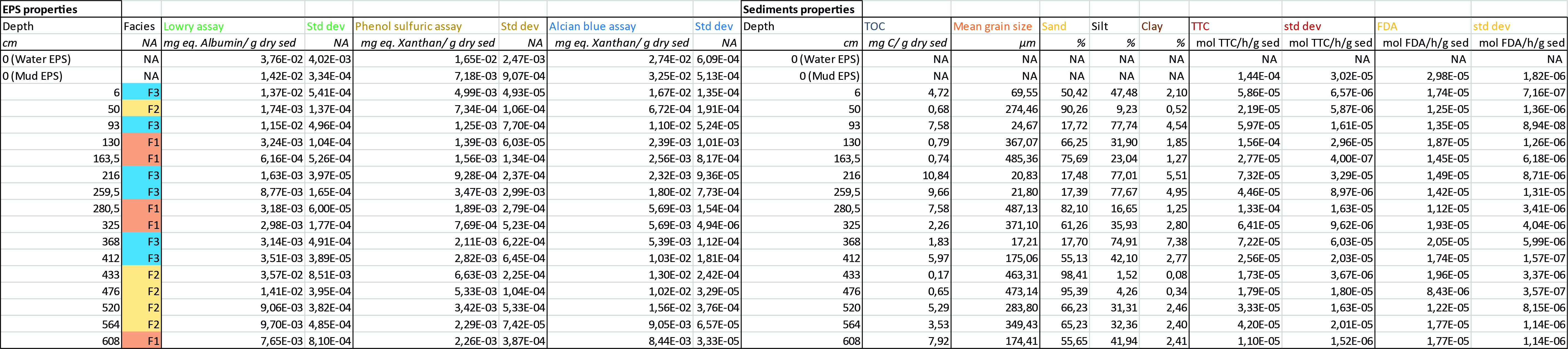

Supplement: SUPPLEMENTARY FIGURE S9 — Principal Components Analysis scatter plot of correlation between the samples from the BXN Long Core. Colors highlighted the main facies found in the core, facies 1 in orange, facies 2 in yellow and facies 3 in blue. [file Image_9.JPEG]
